# Endocannabinoid signaling and epigenetics modifications in the neurobiology of stress-related disorders

**DOI:** 10.1042/NS20220034

**Published:** 2023-07-25

**Authors:** Arthur A. Coelho, Sávio Lima-Bastos, Pedro H. Gobira, Sabrina F. Lisboa

**Affiliations:** 1 Department of Pharmacology, Ribeirão Preto Medical School, University of São Paulo, Brazil; 2 Department of BioMolecular Sciences, School of Pharmaceutical Sciences of Ribeirão Preto, University of São Paulo, Brazil

**Keywords:** Drug abuse, Endocannabinoid System, Epigenetics, Stress

## Abstract

Stress exposure is associated with psychiatric conditions, such as depression, anxiety, and post-traumatic stress disorder (PTSD). It is also a vulnerability factor to developing or reinstating substance use disorder. Stress causes several changes in the neuro-immune-endocrine axis, potentially resulting in prolonged dysfunction and diseases. Changes in several transmitters, including serotonin, dopamine, glutamate, gamma-aminobutyric acid (GABA), glucocorticoids, and cytokines, are associated with psychiatric disorders or behavioral alterations in preclinical studies. Complex and interacting mechanisms make it very difficult to understand the physiopathology of psychiatry conditions; therefore, studying regulatory mechanisms that impact these alterations is a good approach. In the last decades, the impact of stress on biology through epigenetic markers, which directly impact gene expression, is under intense investigation; these mechanisms are associated with behavioral alterations in animal models after stress or drug exposure, for example. The endocannabinoid (eCB) system modulates stress response, reward circuits, and other physiological functions, including hypothalamus–pituitary–adrenal axis activation and immune response. eCBs, for example, act retrogradely at presynaptic neurons, limiting the release of neurotransmitters, a mechanism implicated in the antidepressant and anxiolytic effects after stress. Epigenetic mechanisms can impact the expression of eCB system molecules, which in turn can regulate epigenetic mechanisms. This review will present evidence of how the eCB system and epigenetic mechanisms interact and the consequences of this interaction in modulating behavioral changes after stress exposure in preclinical studies or psychiatric conditions. Moreover, evidence that correlates the involvement of the eCB system and epigenetic mechanisms in drug abuse contexts will be discussed.

## Overview of stress response and circuitry

**Figure 1:**
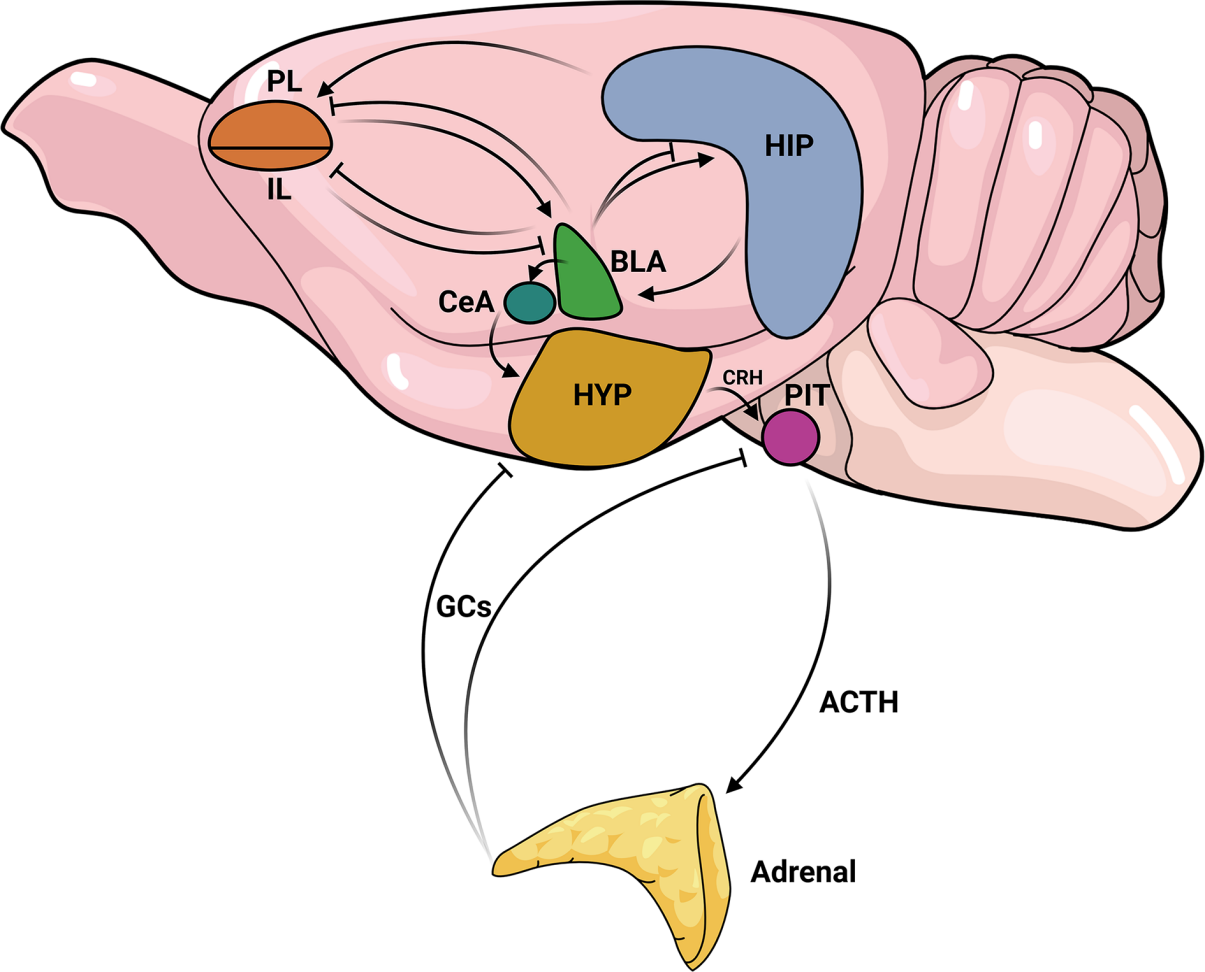
A simplified view of the key brain regions involved in the stress response The amygdala (basolateral-BLA and central-CeA nuclei) is a key region in initiating the stress response. It is an input region for projections sent by other regions, particularly by different subdivisions of the medial prefrontal cortex (mPFC). Specifically, the prelimbic region (PL) of the mPFC exerts excitatory influence on amygdala. In contrast, the infralimbic region (IL) inhibits the basolateral amygdala (BLA). The hippocampus (HIP) contributes to stress response by providing contextual information, with excitatory projections to the PL-mPFC and BLA. The BLA, in turn, inhibits the PL, IL, and has both inhibitory and excitatory projections to the HIP. The balance of these two projections controls the parallel HIP outputs. Integrating this information, the BLA activates the amygdala’s output region, the CeA, which sends excitatory projections to the hypothalamus (HYP), stimulating the production and release of corticotropin-releasing hormone (CRH). CRH acts on the pituitary gland triggering the synthesis and release of adrenocorticotropic hormone (ACTH). ACTH, in turn, is released in the blood and stimulates the synthesis of glucocorticoids (GCs) in the adrenal gland. Finally, released GCs establish a negative feedback loop within the hypothalamus and pituitary, modulating the peripheral and central nervous system areas to facilitate adaptive responses to stress. Depending on the duration and type of stressors, increased amygdala activity, cortical dysfunction and hippocampal atrophy can occur, impairing the ability of this adaptive response to occur, contributing to psychiatric disorders.

Stress is an important and evolutionarily conserved response that modulates several central nervous system (CNS) regions and the endocrine system to prepare the organism to face a challenging experience. The stressors, stimuli that trigger the stress response, can be chemical, physical, psychological, or combinations of these; and they can recruit different brain regions, which could overlap depending on the circumstances [1]. In general, stress exposure activates the hypothalamus–pituitary–adrenal (HPA) axis, leading to release of glucocorticoids (GCs) by the adrenal gland. GCs promote their stress effects mostly by activating glucocorticoid receptors (GR) in the periphery and in the brain, altering the expression of several genes to promote adaptation [2,3]. A simplified view of key brain regions involved in stress response is depicted in Figure 1. However, as reviewing this topic is beyond the scope of this study, the reader can find more comprehensive reviews about this topic, with figures summarizing the current knowledge, in several recent review studies [1,4–7]. Here, we will present some critical information about the central regions affected by stress, that influence behavioral response: the prefrontal cortex, amygdala, and hippocampus.

The prefrontal cortex (PFC) is responsible for complex functions such as integrating and processing different stimuli for decision-making, goal-directed behaviors, and working memory [8,9], in addition to emotional processing. The medial portion of the PFC (mPFC), the most studied in studies related to aversion and stress [10], is mainly divided into the prelimbic (PL) and the infralimbic (IL) portions, which can have distinct or opposite functions [11,12] due to their partially distinct connections and projections [13,14]. In general terms, the PFC is greatly activated during acute stress, and the excess of glutamate release and its impaired reuptake mediated by GCs may result in neurotoxicity [15], proinflammatory factors release, and neuronal death [16,17]. These changes can be related to the neuronal atrophy observed in the PFC of depressed and PTSD patients and animal models involving stress exposure [18,19] which could result in weakened PFC projections to the amygdala and hippocampus, impairing modulatory function exerted by PFC on these brain regions [20,21].

The amygdala is also an essential structure for emotional processing [5] and is divided into different nuclei. The basolateral nucleus (BLA) integrates and processes aversive/stressful stimuli received by several brain regions, being greatly activated during stressful events [5] and fear memory conditioning acquisition and consolidation [22,23]. BLA sends several outputs to the central nucleus (CeA) and the medial nucleus (MeA), which in turn project to other brain areas that will be responsible for triggering adaptative behaviors to facing stress [5]. As mentioned before, the amygdala receives inhibitory projections from the IL mPFC, which can be weakened after stressful situations, resulting in a lack of inhibition of BLA by the PFC and hyperactivation of the amygdala [24,25]. For instance, depressed and PTSD patients have bigger and more active amygdala than healthy volunteers [26–28].

Finally, the hippocampus, which is connected to the amygdala and PFC [29], comprises the dentate gyrus (DG) and Ammon’s horn/Cornus Ammonics (CA) subfields 1, 2, and 3. The CA1 region has outputs to several regions, including the PL and IL mPFC and BLA [30–32]. The hippocampus plays an important role in memory acquisition and discrimination of aversive contexts [33,34]. It also inhibits excessive activation of the HPA axis during stress response [35]. However, stress exposure can cause hippocampus atrophy and decreases neuronal plasticity, impairing its function and resulting, for example, in fear extinction learning deficits and fear generalization [33,36], anxiety-like and depressive-like behaviors [37]. Moreover, decreased hippocampal volume is reported in anxiety disorders, PTSD, and depression in humans [38–41]. Interestingly, based on a monozygotic twins study, it was suggested that a smaller hippocampal volume could be a risk factor for developing PTSD after traumatic events rather than a consequence of PTSD [42].

Although many other brain areas can be involved, it is well-known that a proper synchronization between the amygdala, mPFC, and hippocampus is necessary for adequate stress response and fear processing mechanisms. Therefore, impairment in this circuitry is frequently observed in psychiatric disorders. This poor connectivity can be associated with impaired molecular mechanisms in those brain regions, including dysfunctional neurotransmitter systems, such as endocannabinoids, and epigenetic modifications, which could control neuroplastic changes resulting in long-lasting consequences, such as impaired behavior.

## The (endo)cannabinoid signaling in stress response

The endocannabinoid (eCB) system, activated by stress exposure, is considered a stress-buffer system and essential for several physiological conditions. The main eCBs are anandamide (AEA) and 2-arachidonoylglycerol (2-AG), which are produced on demand post-synaptically in response to an increase in neuronal activity, by actions of N-acyl phosphatidylethanolamine-specific phospholipase D (NAPE-PLD) and diacylglycerol lipase (DAGL) on membrane phospholipids, respectively. These eCBs are released in the synaptic cleft and act retrogradely in cannabinoid receptors (CB_1/2_) or post-synaptically in CB_2_, or in glial cells. CB_1/2_ are G_i_-coupled receptors, then their activation result in the inhibition of adenylyl cyclase, the protein kinase A (PKA), and cellular Ca^2+^ influx, culminating in a blockade in neurotransmitter release that was increased before. Finally, AEA will be degraded by the fatty acid amide hydrolase (FAAH) enzyme mostly in the postsynaptic neuron, whereas 2-AG will be metabolized by the monoacylglycerol lipase (MAGL) enzyme in the presynaptic neuron [43].

Contrasting to CB_1/2_ receptors, the vanilloid receptor 1 (TRPV_1_) is expressed both pre- and post-synaptically. TRPV1 can be activated by AEA, increasing the Ca^2+^ influx and neurotransmitter release. Pieces of evidence suggest that AEA has its actions preferentially via CB_1_ receptors, whereas, at higher concentrations, the predominant effect could reflect its effects on TRPV_1_. This is one of the possible explanations for the bell-shaped dose-response curve often seen with AEA in behavioral responses [44]. It has been shown, for example, that by inhibiting CB_1_ receptors with an antagonist, the extinction learning process is impaired [45,46], and the treatment with a TRPV_1_ receptor agonist has a similar effect [47]. Moreover, the administration of a FAAH inhibitor facilitated the extinction learning process in wild-type mice [46]. Finally, the facilitation of CB_1_ signaling by a drug that blocks FAAH and antagonizes TRPV_1_ receptors is more potent in blocking the expression of fear conditioning than inhibiting these two targets individually, supporting the opposite role for CB_1_ and TRPV_1_ receptors in triggering fear behavior [48].

Overall, a very reductionist description of the eCB system is described here. In the past few years, several other targets of the eCB system were described, such as the G-protein-coupled receptor 55 (GPR55) and the peroxisome proliferator-activated receptor γ (PPARγ), which interact with eCBs and other substrates, promoting different effects. A more recent detailed review of this topic can be found in [43].

The eCB system is altered in several pathological conditions, such as cancer, gastrointestinal and cardiovascular diseases, eating disorders [49], and stress-related psychiatric conditions [39,40,50,51]. For instance, studies have shown that depressed women have decreased serum levels of AEA and 2-AG [52]. Moreover, depressed suicide victims have increased expression of CB_1_ receptors in the dorsolateral PFC [53]. Finally, genetic studies have shown that polymorphisms of the CB_1_ receptor have greater prevalence in depressed and anxious patients [54,55] and are correlated with treatment-resistant depression [56]. Interestingly, pharmacological studies indicate that the administration of rimonabant, a CB_1_ antagonist, increases symptoms of depression and anxiety in healthy individuals [57] and that the effects of conventional antidepressant drugs depend on the eCB signaling [58].

In PTSD patients, reduced peripheral AEA levels and higher expression of CB_1_ receptors in the brain are also reported [59]. Interestingly, non-PTSD individuals carrying the C385A allele of the rs324420 polymorphism, a mutation of the FAAH gene, had increased AEA levels, enhanced fear extinction [60], and lower amygdala reactivity during fear extinction recall [61]. Also, polymorphisms of the CB_2_ receptor and FAAH enzyme have been associated with greater susceptibility to childhood trauma and the development of psychiatry disorders later in life [62,63]. Other polymorphisms in the eCB system, including in the CB1 receptor, are associated with fear extinction and/or PTSD [64–66].

Altogether these data suggest a strong relationship between the eCB system and psychiatry conditions. Based on the existing evidence, impaired eCB signaling in the brain, induced by stress or as consequences of polymorphisms, could be involved in the neurobiology of stress-related disorders, including depression and PTSD. Therefore, targeting these changes in the eCB system is a potential tool to improve the outcome of those disorders, potentially achieving remission in treatment-resistant patients. However, this still need further investigation. More about these discussions can be found in excellent recently published reviews [64,67,68].

Animal model studies also corroborate the involvement of eCB signaling in the development of behavioral changes related to psychiatric symptoms. For instance, CB_1_ KO mice have elevated levels of depression- and anxiety-like behaviors after stress [69–72]. Moreover, these mice also have impaired fear extinction, a key feature of PTSD animal models [73]. Pharmacologically, these behavioral alterations are reproduced after chronic administration of CB_1_ antagonists [74,75]. CB_1_ agonists have also been shown to have antidepressant and anxiolytic effects after stress [76–78]. Furthermore, several papers reported the participation of the eCB system, particularly CB_1_ receptors, promoting fear extinction facilitation in different animal models [45,46,79,80]. CB_2_ participation in promoting behavioral responses seems to be more complex. The overexpression of this receptor promoted reduced levels of anxiety-like behaviors in the light-dark box and elevated plus maze in mice [81]. However, evidence shows that chronic administration of a CB_2_ antagonist produces an anxiolytic effect [82]. Another study from the same group observed that the overexpression of CB_2_ receptors reduced depressive-like behaviors in mice, whereas the administration of AM630, a CB_2_ antagonist, induced an antidepressant effect in wild-type mice. The drug had no effect in the transgenic line [83]. Moreover, CB_2_ KO mice have impaired contextual, but not cued, fear conditioning and enhanced spatial memory [84]. More studies using genetic and molecular techniques with specific cell types, such as microglia and astrocytes, are needed to investigate the interaction of CB_2_ expression and activity with other signaling systems involved in mood regulation and behavior.

Multiple mechanisms involved in the physiopathology of psychiatric conditions, like major depression, anxiety, and PTSD, can be regulated by the eCB system. Therefore, the resultant effects from eCB system manipulation have been related to several molecular alterations. For example, eCBs can counteract dysregulation in neurotransmitter systems [85–87], promote neuroplasticity [88,89], and attenuate inflammatory effects [90–93] induced by stress. In mice, both genetic deletion and the antagonism of CB_1_ receptors in the mPFC prolonged CORT release after stress. It has been proposed that the activation of CB_1_ in mPFC GABAergic interneurons disinhibits excitatory neuronal projections that are responsible for terminating stress response [94].

A more recent proposal regarding stress consequences implies the modulation of epigenetic mechanisms in the brain to promote behavioral changes. This review will focus on the crosstalk between the eCB system and epigenetics mechanisms to modulate the stress response. Considering a general knowledge about epigenetic mechanisms are necessary to understand how they can impact the eCB system, in the next section we will give an overview about this knowledge.

## Key epigenetic mechanisms in stress response

Epigenetics focuses on the interaction between the environment and genome, whereby gene expression is modulated in response to new stimuli. In the short or long term after stress experiences, several epigenetic marks can be modified to adapt the organism to the environment; these marks can even be transmitted between generations. Because of a maladaptive response, these epigenetic changes can predispose the organism to diseases. There are three major epigenetic mechanisms for the regulation of gene expression: DNA modifications, histone modifications, and interference RNAs. Below, we will briefly describe each one of them, associating them with modifications in important stress-related systems.

### DNA methylation

The DNA is passive to chemical modifications that do not change the nucleotide sequence but regulate gene transcription. Among many modifications described in the literature, cytosine methylation at the 5′ position (5mC) is the most investigated. The DNA-methyltransferases (DNMTs) family of enzymes in mammals, comprising DNMT1, DNMT3a, and DNMT3b, catalyzes the reaction of a methyl group addition to a cytosine [95]. The reaction occurs mainly in CpG dinucleotides, distributed throughout the genome but more concentrated in CpG islands in promoter regions of the genes. 5mC is recognized by methyl-CpG binding proteins (MBPs), such as methyl-CpG binding protein 2 (MeCP2), commonly related to psychiatry diseases [96,97]. 5mC is usually associated with the repression of gene expression, but it also can promote gene expression activation depending on the location of the CpG island in the gene body [98]. Despite some stability, DNA methylation is a dynamic process constantly subject to reversal (demethylation) by active and passive forms. The passive form occurs through DNA damage or replication, while the active form is a process based on methyl-cytosine modifications through the ten–eleven translocation (TET) enzyme family and the activation-induced cytidine deaminase/apolipoprotein B mRNA-editing enzyme complex (AID/APOBEC) [98].

Both DNA hyper- and hypomethylation of stress-related genes are found in various neuropsychiatric and neurological diseases [99–102]. Most clinical studies focus on genes related to glucocorticoid response and serotonin neurotransmission [100,103]. The promoter region of the glucocorticoid receptor gene (NR3C1) appears to be extremely sensitive to DNA methylation. Various stressor events, including child abuse, war and genocide-related trauma, maternal depression, or violence during pregnancy, correlate with increased [104–110] and decreased [105,111,112] methylation levels in NR3C1. Methylation of NR3C1 is also altered in the post-mortem brains of suicide victims and is associated with childhood traumatic experiences [113,114]. In these works, methylation levels frequently are inversely proportional to NR3C1 expression, and low levels of GR can directly impact the feedback of glucocorticoid release and HPA activity, contributing to altered responses to stress. Regarding the serotoninergic system, the serotonin transporter gene (SLC6A4) methylation levels are related to traumatic events, child abuse, work stress, and depression [115–121], and although it does not correlate with mRNA expression in the blood of patients, the hypomethylation of SLC6A4 is proposed to be a biomarker of diagnosis and drug response to major depression [118,122].

### Histones modifications

Histones are proteins that, together with DNA, make the chromatin and organize its packaging state. There are four types of histones: H2A, H2B, H3, and H4. The addition of chemical groups to amino acid residues of the histones alters their binding to DNA modulating the access of transcription factors. The addition of group acetyl, or acetylation, is a modification primarily associated with gene expression. It occurs due to the action of histone acetyltransferases (HATs) and is erased by histone deacetylases (HDACs). Histone methylation is another modification related to gene expression or repression depending on the location of the methyl group. It is catalyzed by histone methyltransferases and reversed by histone demethylases, also occurring on lysine residues [123,124].

In humans, there are only a few works exploring histone mark changes in the context of stressful experiences and neuropsychiatric diseases. In these studies, they found differences in tri-methylation of H3 at lysine 27 (H3K27me3) and at lysine 4 (H3K4me3) levels in the brains of suicide victims when it was compared between control and depressive groups [125–127]. On the other hand, in animal models, several types of stressors, such as maternal separation, social stress, restraint stress, and chronic mild stress, induce alterations in HDACs and histone acetylation/methylation levels in a global or gene-specific manner [128–130]. Interestingly, antidepressant drugs with different mechanisms of action, such as ketamine, imipramine and fluoxetine, not only ameliorate behavior alterations after the stress but also alters HDACs activity and expression, or impact levels of acetylation at specific histone residues associated with important genes related to synaptic plasticity, such as *Nr2b* and *Bdnf* genes [131–138].

### Non-coding RNAs

Micro RNAs (miRNAs), small interfering RNAs (siRNAs), long non-coding RNAs (lncRNAs), and piwi RNAs (piRNAs), along with others, are regulatory non-coding RNAs, which regulate transcription and translation processes most often through binding to mRNA [139]. Among these, miRNAs are the most characterized regarding their biogenesis and role in diseases. The binding of miRNA to complementary sequences of mRNAs induces their cleavage or, in the case of partial complementarity, induces the inhibition of translation in ribosomes [139].

miRNAs can either be regulated by stress signaling or act as a regulator of the stress response [140]. The capacity of a single miRNA to bind several mRNAs allows it to modulate entire cellular pathways, which in part explains altered levels of the same miRNA in different diseases. On stress-related diseases, it is possible to highlight some stress-regulated genes such as *Nr3c1, Bdnf, Ntrk2, Slc6a4*, and *Crhr1* [141–144], which are also found to be regulated by histone modifications and DNA methylation [145–147]. Moreover, the miRNA network regulates and is regulated by proteins related to other epigenetic processes, such as HDACs, DNMTs, and MeCP2 [148]. Due to the stability and the facility to detect changes in easily accessible tissues, such as blood and saliva, miRNAs are indicated as excellent biomarker candidates for disease and treatment responsiveness [149].

## Possible crosstalk between the eCB system and epigenetic mechanisms in stress response and psychiatric disorders

### (Endo)cannabinoid control of epigenetic mechanisms

*Cannabis* use induces epigenetic alterations, supporting a relationship between the eCB system and epigenetic mechanisms. For instance, *Cannabis*-dependent patients have reduced methylation of the CB_1_ receptor gene promoter (*Cnr1*) and increased CB_1_ expression in the blood [150]. The same profile of methylation and CB_1_ expression was observed in peripheral blood lymphocytes of patients with schizophrenia reporting the use of *Cannabis* [151]. Moreover, prenatal *Cannabis* exposure decreased D_2_ receptors mRNA in Nucleus Accumbens (NAc) and amygdala of aborted fetuses, which was replicated in an animal model. In this model, there was increased di-methylation at lysine 9 of H3 (H3K9me2), a repressive mark, and decreased H3K4me3, mentioned earlier, an enhancer mark, and RNA polymerase II at the *Drd2* gene locus [152,153], supporting the role of an epigenetic mechanism induced by *Cannabis* in decreasing D_2_ expression. These changes have implications for drug addiction, which will be discussed later, and other psychiatric conditions. For instance, the chronic administration of a CB_1_ agonist to adolescent male rats has been implicated in greater susceptibility to stress and anxiety-like behavior, in addition to increase DNMT and global methylation levels in the PFC of their adolescent offspring [154]. Also, paternal activation of CB_2_ receptors was implicated in impaired offspring growth via reduced expression of TET enzymes and altered DNA methylation in several genes [155].

Regarding treatment with CBD, it was demonstrated that acute CBD treatment decreased immobility in mice in the forced swimming test, similar to what was observed with DNMT inhibitors (5-AzaD and RG108). Interestingly, the combination of ineffective doses of CBD and DNMT inhibitors induced similar antidepressant effects, suggesting CBD effects could be directly modulating DNMTs. In fact, all drugs prevented the swimming stress-induced reduction of the DNA methylation in the PFC and the increase in the hippocampus. However, whereas the DNMT activity was decreased by swimming stress in the PFC and increased in the hippocampus, CBD could only counteract the first in this work [156]. In contrast, the hippocampal neurodegeneration induced by iron administration in neonatal rats, which induces mitochondrial DNA methylation alterations, was reverted by treatment with chronic CBD during adulthood [157]. Finally, subacute treatment with CBD induced hypomethylation of DNMT3a in the mouse hippocampus [158], a mechanism already shown to induce gene expression related to neurogenesis [159,160]. These pieces of evidence suggest a role for DNA methylation in CBD effects in animal stress models. As described before, many of these makers are also involved in stress-related disorders, and CBD has anxiolytic/antidepressant effects in psychiatric patients [161]. Therefore, it is possible to suggest that these CBD effects may involve DNA methylation; however, there are no studies in humans with this analysis, which would be very useful for better conclusions.

Histone modifications may also be involved in effects mediated by cannabinoids. Repeated co-administration of THC and CBD increased the acetylation in lysine 9 (H3K9ac) and 14 (H3K14ac) of H3 in the ventral tegmental area of adult mice [162]. In another study, acute CBD treatment increased levels of methylation and acetylation markers H3K4me3, H3K27me3, and H3K9ac in the cerebral cortex. In contrast, it decreased H3K9ac levels in the hypothalamus and H3K4me3 in the pons in rats, demonstrating that its effects are brain area-specific [163].

Chronic unpredictable stress (CUS) increased the nuclear expression and activity of HDAC2 and decreased the expression of CB1 levels, mainly in glutamatergic neurons, in the mouse cingulated cortex. Moreover, CUS reduced the expression of H3K9ac associated with CB_1_ and Neuropeptide Y (NpY) genes. They also showed that URB597, a FAAH inhibitor, which is expected to increase anandamide levels, reverted stress effects in the *Npy* gene, but not in *Cnr1*, and anxious behavior [164]. Like stress, a TRPV_1_ agonist (capsaicin), which increased immobility in the forced swimming test, increased HDAC2 expression in the mouse DG of the hippocampus [165] and enriched HDAC2 expression at *Dlg4, Syp, Gria1*, and *Gria2* gene promoters, all related to neuroplasticity [166]. In contrast, genetic deletion of TRPV_1_ receptors, which induced an antidepressant-like phenotype, reduced HDAC2 levels in the same brain region and consequently increased levels of H3 and H4 global acetylation. In addition, TRPV_1_ knockout mice, contrary to what was observed with capsaicin injection, showed increased levels of plasticity and neurogenesis-related genes in the hippocampus, in addition to being resilient to stress [166]. Therefore, considering anandamide activates CB_1_ and TRPV_1_ receptors, we suggest that the bell-shaped profile of anandamide effect on behavior may be mediated by differential effects on CB_1_ and TRPV_1_ receptors, among other mechanisms, regulating the expression and activity of HDAC2, histone acetylation levels, gene expression, and neuroplasticity.

As described before, miRNAs play an essential role in gene expression regulation, being implied in several diseases. eCB system activity, in turn, seems to regulate miRNA expression and, therefore, could impact the pathogenesis and treatment of stress-related diseases. In mice, chronic mild stress (CMS) increased expression of some miRNAs in the PFC (miR-9-5p, miR-128-1-5p, and miR-382-5p) [167] that target *Drd2, Clock, Map2k, Mapk1*, and *Bdnf* genes [168–170], all related to the physiopathology of depression. Moreover, CMS induced decreased expression of others miRNAs (miR-16-5p, miR-129-5p, and miR-219a-5p) [167], which target *Slc6a4, Htr2a, Bdnf, Grm7, Camk2a*, and *Camk2g* genes, which are also related to depression physiopathology and antidepressant response [168,171–174]. In the same study, stressed animals treated with anandamide showed increased expression of all these miRNAs compared with the vehicle group; the depressive-like effect of stress in the forced swimming test was reverted [167]. Moreover, early life stress-induced depressive-like behavior in rats and downregulated miR-16 in males and miR-135a in females in the mPFC. These changes were reversed when rats received a FAAH inhibitor [175].

Additionally, lower expression levels of *let-7d* miRNA were observed in the cortex and hippocampus of CB_1_ knockout mice or after CB_1_ knockdown in zebrafish embryos. Conversely, the knockdown of *let-7d* in zebrafish embryos increased the expression of CB_1_ receptors, suggesting negative feedback in this regulation [176,177]. Moreover, *let-7d* overexpression in adult mouse hippocampus induced anxiolytic- and antidepressant-like effects [178]. Thus, it is arguable that anxiolytic and antidepressant effects induced by CB_1_ activation are promoted by *let-7d* expression and that this mechanism may be impaired in psychiatric conditions, such as depression. Furthermore, the anxiolytic and antidepressant effects induced by *let-7d* increased expression may occur, between other mechanisms, by the negative regulation of dopamine D3 receptors, mu-opioid receptors, TLX, an orphan nuclear receptor, and upregulation of miR-9, regulating neuroplasticity, cellular proliferation, neuronal differentiation, and migration [178–180].

In summary, the eCB system activity regulates the expression and activity of epigenetic enzymes, such as TETs, DNMTs, and HDACs, which result in differential global and specific-site levels of DNA and histone modifications. Moreover, the eCB system is also involved in miRNA expression regulation. All these alterations change gene expression related to neurotransmission, neurogenesis, and neuroplasticity. These mechanisms, also altered by stress, may be involved in the development of psychiatric conditions; therefore, more studies are needed to better understand how they work in physiological and pathological conditions to determine if they could be targets for treating these conditions.

So far, only a few studies combine stress protocols, modulation of the eCB system, and evaluation of epigenetic output, highlighting the need for more studies addressing that combination. These studies are summarized in Table 1. How eCB system molecules modulate epigenetic factors in stress-related contexts are outlined in Figure 1A.

**Table 1:** Studies combining stress protocol, eCB system modulation and epigenetic output in animal models

Pharmacological intervention	Sex, strain, age	Stress	Behavioral outputs	Molecular outputs	Reference
Prenatal CB1 agonist (WIN55212-2; 1.2 mg/Kg/day); from PND30 to PND49	♂ Rats, adolescent and adult	Offspring (PND60) exposed to Unpredictable stress during one week	Anxiogenic effect in the OFT induced by WIN in stressed offspring (PND68)	WIN ↑ global methylation and DNMT3a levels in the PFC of stressed offspring WIN ↑ DNMT1 levels in the PFC only in non-stressed offspring	[154]
FAAH inhibitor (URB-597; 1.0 mg/Kg/day); from the 5th to the 11th week of stress protocol	♂ mouse, 6 weeks old	CUS for eleven weeks	Not evaluated	CUS ↑ expression and activity of HDAC2 in the cingulate cortex CUS ↓ expression of H3K9ac associated with npy and cnr1 genes in the cingulate cortex URB reverted the effect of stress in the npy gene	[164]
TRPV1 KO TRPV1 KD in DG	♂ mouse, 4-5 weeks old	CUS for 14 days	CUS induced learned helplessness (LHT) in WT, but not in TRPV1 KO. Antidepressant- and anxiolytic effect of TRPV1 KO in the FST and NSFT, independent of stress exposure. TRPV1 KD in the DG mimics the TRPV1 KO phenotype in the FST	↓ HDAC2 levels in the hippocampus of TRPV1 KO ↑ H3 and H4 global acetylation levels in TRPV1 KO hippocampus ↑ expression of neuroplasticity and neurogenesis genes in TRPV1 KO hippocampus TRPV1 KD in the DG mimics the TRPV1 KO phenotype	[166]
AEA; 5 mg/Kg/day; After forced swimming pretest, 5 hours before and 1 hour after FST	♂ mouse, 3 months old	CMS for 7 weeks	CMS induced depressive-like behavior in the FST and SPT AEA ↓ the stress effect in the FST	CMS ↑ miR-9-5p, miR-128-1-5p, and miR-382-5p, and ↓ miR-16-5p, miR-129-5p, and miR-219a-5p expression in the PFC AEA ↑ expression of miR-9-5p, miR-128-1-5p, miR-382-5p, miR-16-5p, miR-129-5p, miR-219a-5p in the PFC of stressed mice	[167]
FAAH inhibitor (URB-597; 0.4 mg/Kg/day); From PND45 to PND60)	♂ and ♀ rats	ELS (from PND7 to PND 14)	ELS: ♂: ↓ distance traveled and ↑ freezing time in the OFT; ♀: ↑ freezing time in the OFT; URB treatment: ↑ SP in stressed ♂ and ♀; ↓SP in non-stressed ♀; ↑ the SR discrimination index in stressed ♂ and ♀; ↓ SR in non-stressed ♂; ↓ immobility in the FST in stressed ♂ and ♀; ↑ immobility in FST in non-stressed ♂;	URB treatment: ↑ expression of miR-135a in the mPFC and ↓ it in the LHa and DR of non-stressed ♂ and ♀; Normalized the expression of miR-135a in the mPFC of stressed ♀; ↓ expression of miR-135a in the CA1 region of non-stressed ♂; ↑ the expression of miR-135a in the CA1 region of non-stressed ♀; ↓ miR-135a expression in the DR of stressed ♀; Normalized the expression of miR-16 in the mPFC of stressed ♂; ↑ expression of miR-16 in the mPFC of non-stressed ♂ ↑ expression of miR-16 in CA1 region of non-stressed ♀ ↑ expression of miR-16 in the LHa of stressed ♂ ↓ expression of miR-16 in the LHa of non-stressed ♀; ↑ expression of miR-16 in the DR of stressed ♀;	[175]

↑: increased; ↓: decreased; ♀: female animals; ♂: male animals; AEA, anandamide; CA1, Cornus Ammonics subfield 1 of hippocampus; CB_1_ and CB_2_, cannabinoid type 1 and 2 receptors; CMS, chronic mild stress; CUS, chronic unpredictable stress; DG, dentate gyrus; DNMT, DNA-methyltransferase; DR, dorsal raphe; ELS, early life stress; FAAH, fatty acid amide hydrolase; FST, forced swimming test; HDAC, histone deacetylase; KD, knockdown; KO, knockout; LHa, lateral habenula; LHT, learned helplessness test; mPFC, medial prefrontal cortex; NSFT, novelty suppressed feeding test; OFT, open field test; PFC, prefrontal cortex; PND, postnatal day; SP, sucrose preference; SPT, sucrose preference test; SR, social recognition; TRPV_1_, transient receptor potential vanilloid type 1.

### Epigenetic control of the endocannabinoid system

Although the relevance of the epigenetic mechanisms to the activity of the endocannabinoid system is well known [181–184], this regulation in the context of stress is less explored. The investigation of epigenetic control of the eCB system relies mainly on the regulation of *Cnr1* and *Faah* genes. This specificity can be explained by the attention these two genes receive due to their pharmacological importance in physiology and disease and their susceptibility to being regulated by epigenetic marks.

DNA methylation levels of *Cnr1*, for example, is reported to be inversely proportional to mRNA and protein expression of the gene [185–191]. *Cnr1* methylation pattern is recurrently found altered in a variety of situations, such as diet [190,192,193], patients with schizophrenia [189], and THC consumption [150]; *Cnr1* is also susceptible to demethylation by the agent 5-aza-2-deoxycytidine [194]. On the other hand, *Faah* hypermethylation is associated with alcohol consumption [195], while hypomethylation, along with an increase in mRNA and protein expression, is related to Alzheimer’s disease patients [196]. Nonetheless, the methylation of *Cnr1* and *Faah* in stressful conditions are less explored. Chronic stress induces depressive-like behavior and results in hypermethylation in *Cnr1* [188], including in several CpG islands of *Cnr1* gene in sperm of the stressed rats and in their offspring’s brains [197]. In PTSD patients, the *Cnr1* gene was one of several uniquely methylated genes found in patient’s PBMC [198], suggesting variations in CB_1_ expression could be involved in the pathology of this disease, as well as other psychiatric diseases, as discussed in previous reviews [67,199].

Histone modifications are also found in *Cnr1* and *Faah* genes. Ethanol treatment in mice is correlated with an increase in histone acetylation marker H4K8a and *Cnr1* expression and a decrease in histone methylation marker H3K9me2 in the neocortex and hippocampus [200,201]. On the other hand, histone methylation marker H3K9me2 and mRNA expression of the *Cnr1* gene are induced at dorsal root ganglion in a model of nerve injury in mice [202]. Although no change in DNA methylation was observed in a model of binge-eating behavior, histone acetylation H3K9ac associated with the *Faah* gene and its mRNA expression decreased after frustration stress [203]. Moreover, after exposure to CUMS, histone acetylation H3K9ac decreased in the *Cnr1* gene, although its mRNA expression remained unchanged [164].

Several miRNAS are reported as modulators of genes of the eCB system, including genes of *Cnr1, Cnr2*, and *Faah* (Table 2). Except for the miR-let-7d, which inhibits but does not have *Cnr1* as a direct target [176], all described miRNAs have predicted pairing to their targets so far [176,192,204,205,206,207,208,209,210,211]. All these miRNAs directly bind to the transcript, inhibiting eCB-related gene expression.

**Table 2:** miRNAs related to the eCB system in animal models involving stress exposure or in psychiatric patients

Gene	miRNA	Population/Model	Sample	Results	Reference
***Cnr1***	MiR23a [192]	CSD susceptible mice	Hippocampus	↑	[212]
	miR-29a [204]	MS-ARS rats	mPFC	↑	[213]
		Treatment-resistant depression	Serum	↓	[214]
		Chronic academic stress students	Total blood	↑	[215]
		ARS mice	Basolateral amygdala	↑	[216]
	miR-29b [205]	MDD patients	Cerebral spinal fluid	↑	[217]
		CUMS mice	PFC	↓	[218]
		ARS mice	FC	↑	[219]
		Fear conditioning mice	Hippocampus	↓	[220]
	miR-30b [206]	MDD suicide subjects	PFC	↑	[221]
		ARS mice	FC	↑	[219]
		CSD rats	Ventral hippocampus	↑	[222]
		CUMS resilient mice	Amygdala	↓	[223]
		Fear conditioning mice	Hippocampus	↓	[220]
	miR-128 [207]	PTSD patients	Total blood	↓	[224]
		Tail shocks rats	Amygdala	↑	[225]
		Fear conditioning mice	PFC	↑	[226]
		Fear conditioning mice	Hippocampus	↑	[220]
		MDD patients after escitalopram treatment	Total blood	↑	[146]
		Depressed suicide subjects	Amygdala	↑	[225]
	miR-301ª [192]	Depressed suicide subjects	PFC	↓	[227]
		CUMS mice	Ventral tegmental area	↓	[228]
	miR-338-5p [208]	Psychological stress susceptible mice	PFC	↑	[229]
		CUMS resilient mice	Amygdala	↓	[223]
	miR-494 [209]	Depressed suicide subjects	PFC	↓	[227]
		MDD patients	Plasma	↑	[230]
		Ethanol exposed rat overexpressing antagomiR-494	Amygdala	Anxiolytic	[231]
		MDD patients after escitalopram treatment	Total blood	↑	[146]
		MDE patients	Peripheral blood mononuclear cells	↑	[232]
		PTSD Rat model	Serum	↑	[233]
		ARS mice	FC	↓	[219]
	miRNA let-7d [176]	ARS mice	PFC	↓	[234]
		MDD patients	Total blood	↓	[235]
		MDD patients after escitalopram treatment	Total blood	↓	[146]
		PTSD mice model	PFC	↓	[236]
			Hypothalamus	↑	[236]
		Mice overexpressing miRNA let-7d	Hippocampus	Anxiolytic	[178]
				Antidepressant	[178]
		CUMS resilient mice	Amygdala	↑	[223]
		Fear conditioning mice	Hippocampus	↓	[220]
***Cnr2***	miR-187-3p [210]	ARS mice	Basolateral amygdala	↓	[216]
		Contextual fear conditioning mice	Dorsal hippocampus	↓	[237]
		Extinction of contextual fear conditioning mice	Basolateral amygdala	↑	[238]
		Psychological stress susceptible mice	mPFC	↓	[229]
		CUMS susceptible mice	Amygdala	↓	[223]
	miR-665 [209]	CUMS resilient mice	Amygdala	↑	[223]
***Faah***	mir-411 [211]	MS rats	Hypothalamus	↑	[239]
		MS rats	PFC	Inversely proportional to sucrose preference	[240]
		CUMS rats	Hippocampal Dentate Gyrus	↑	[241]

↑: miRNA up-regulation; ↓: miRNA down-regulation; ARS, acute restraint stress; CRS, chronic restraint stress; CSD, chronic social defeat; CUMS, chronic unpredictable mild stress; MDD, major depressive disease; mPFC, medial prefrontal cortex; MS, maternal separation; PFC, prefrontal cortex, PTSD, post-traumatic stress disorder.

Similar to what is seen in studies evaluating DNA methylation and histone modifications related to the eCB system, most works describe miRNAs regulating *Cnr1* expression in the context of stressful or psychiatric conditions. For instance, miR-128 is down-regulated in the blood of PTSD patients [224] but is up-regulated in the brain of depressed subjects [225]; however, it is also reported to increase in blood after 12 weeks of antidepressant treatment [146]. Overall, these data could suggest that miR-128 participates in disease and treatment response, but this pattern can be different depending on the psychiatric condition and the evaluated tissue. In animal models, miR-128 up-regulation is found in the amygdala, PFC, and hippocampus of stressed mice [220,225,226], which indicates it can participate in brain functions. Another miRNA, miR-301a, is also up-regulated in the brain of depressed suicide victims [227] and chronically stressed rats [228]. miR-494 findings in the blood and brain are contrasting: it was upregulated in blood of major depression patients [235] and after antidepressant treatment [146], in depression episodes [232], in a PTSD rat model [233]; however, it was downregulated in the brain of depressed suicide victims [227] or in the brain of acutely stressed rats [219]. miR-494 also had an anxiolytic effect in ethanol-exposed rats [231]. Moreover, miR-29a is up-regulated in the blood of stressed students but down-regulated in treatment-resistant depression patients [214,215]. After restraint stress, rats subjected or not to maternal separation have up-regulation of miR-29a in the amygdala and PFC [216], which is increased in the cerebral spinal fluid of MDD patients [217]; it is also increased in the frontal cortex of mice exposed to acute restraint stress [219] but decreased in the frontal cortex after chronic stress [218] and in the hippocampus 1 h after footshock stress in a fear conditioning paradigm [220]. Similarly, 1 h after fear conditioning, there was also a reduction in the miR-30b expression in the hippocampus [220], as seen after acute restraint stress [219], whereas chronic stress increases the same miR-30b in the hippocampus [223]. Another miRNA, miR-let-7d, appears to be important in various stress processes. It was reduced in the blood of MDD patients, and increased after antidepressant treatment [146,235]; its levels changed in the PFC, hypothalamus, hippocampus, and amygdala of animal models after different types of stressors [220,223,234,236]. Meanwhile, overexpression of miR-let-7d in the hippocampus has anxiolytic and antidepressant effects in mice, corroborating its function in behavior and potential impact in neuropsychiatry diseases [178]. Although there is no evidence of alterations in humans, miR-338-5p and miR-23a are altered after protocols of social stress and chronic unpredictable stress [212,223,229]. These works evidence a complex control by different miRNAs, including similarities or differences among patients with different psychiatric conditions and differences in animal models involving stress exposure.

There are two miRNAs reported to modulate *Cnr2* in animal models of stress: miR-187-3p and miR-665. miR-187-3p is regulated in several stress protocols, including chronic mild stress, acute restraint stress, psychological stress, and after fear conditioning, indicating that miRNA as having an important role in stress responses in general [216,223,229,237,238]. Both acute and chronic stressors can decrease miR-187-3p expression in the amygdala, while it was up-regulated after the evaluation of extinction of conditioned fear memory [238]. Additionally, miR-665 is altered in the amygdala, being up-regulated after chronic mild stress [223]. miR-411, for the best of our knowledge, is the only FAAH miRNA regulated by stress, and it is only found regulated in animal models; it was increased in the hypothalamus, PFC, and hippocampus after maternal separation or chronic unpredictable mild stress [239–241].

eCB system genes are considerably sensitive to epigenetic control, particularly under stressful experiences, although the mechanisms are not completely elucidated. The discussed evidence highlights the importance of epigenetic mechanisms in the eCB system response to stress and in its dysfunction. For instance, histone modifications are fundamental to memory consolidation and extinction [242,243], and intervention in these processes could be key to treating trauma-related disorders. In fact, some stressors can induce histone modifications in the *Faah* and *Cnr1* genes, which can reverberate or not in mRNA expression; moreover, the histone modifications, as acetylation, is one of the proposed mechanisms for the action of antidepressant drugs [244]. Even when the gene or the protein expression is not altered, epigenetic markers can influence the gene expression pattern in response to the environment. DNA methylation and miRNA expression are already suggested as biomarkers of disorders, prognosis, treatment prediction, and response. Considering the findings with the CB_1_ receptor in human and animal models, epigenetic modifications in the *Cnr1* gene are promising biomarkers in neuropsychiatry conditions [67].

## Possible cross-talk between eCB system and epigenetics in drug abuse and stress

The eCB system is critical to the reward-related effects of dopamine, which is involved in the neurobiological mechanism underlying drug addiction [245]. Indeed, the modulation of the eCB system regulates molecular and behavioral responses promoted by distinct addictive drugs, including psychostimulants and alcohol [246,247]. Stress is an important risk factor in the neurobiology of drug addiction [248,249]. Previous stress exposure is correlated to the vulnerability to developing the disorder and the reinstatement of drug seeking. Interestingly, behavioral and molecular evidence indicates that the eCB system is a required element in the ability of stress to modulate drug responses [250,251]. This convergence is consistent with the fact that exposure to addiction drugs promotes changes in important brain structures also involved in stress biology, such as the PFC, nucleus accumbens, hippocampus, and amygdala, which are also important targets for cannabinoids [252]. In this way, similarly to what was described for stress events, exposure to addiction drugs also modulated the eCB system, which involves epigenetic mechanisms.

For instance, cocaine self-administration (SA) promotes histone modifications and chromatin looping in the eCB system-associated genes [253]. Animals exposed to cocaine demonstrated increased H3K4me3 enrichment on the hippocampus’s promotor regions of FAAH and DAGLα coding genes. Moreover, using a 4C-seq approach targeting the *Cnr1* promoter, authors also demonstrated that cocaine SA induces remodeling of chromatin loops in the hippocampus and the NAc, suggesting that 3D chromatin architecture at the *Cnr1* locus was substantially changed following cocaine exposure [253]. Pieces of evidence also have demonstrated that the eCB system undergoes epigenetic modulation by alcohol, as briefly mentioned before. A blind epigenome-wide analysis of datasets that explored hazardous drinkers and binge drinkers versus controls evidenced that *Faah* hypermethylation is associated with alcohol consumption [195]. Accordingly, an elevation in the expression of CB_1_ associated with increased H4K8ac at the *Cnr1* promoter was observed in adult mice exposed to alcohol on postnatal day 7 (PD7) [254]. These results provide evidence that epigenetic mechanisms contribute to altered regulation of the eCB system in response to specific abuse drugs.

Interestingly, epigenetic changes promoted by exposure to alcohol also appear to be modulated by the eCB system. Nagre and colleagues observed that treatment with ethanol in PD7 mice impaired DNA methylation through reduced DNA methyltransferases (DNMT1 and DNMT3A) levels; these effects were reversed by the blockade of CB_1_ before ethanol treatment [255]. Similarly, alcohol exposure at the PD7 was associated with enhanced HDAC1, HDAC2, and HDAC3 expression, which was also prevented by administering a CB_1_ receptor antagonist before alcohol exposure [256]. Moreover, using a similar protocol, another study demonstrated that exposure to ethanol activates the apoptotic caspase-3 enzyme via CB_1_ in neonatal mice and causes a reduction in MeCP2 levels [257]. Regarding miRNA processes, a reduction in the expression of brain CB_1_ was coupled with an increased complementary miR-26b in a mouse model of fetal alcohol spectrum disorders [258].

Corroborating the role of the eCB system in the regulation of drug response during development, converging pieces of evidence support that treatment with THC in early phases of development promotes epigenetic changes [259]. For instance, prenatal THC exposure significantly modifies the histone methylation profile in the NAc. Subjects exposed to THC during prenatal stage showed a decreased level of the H3K4me3 [152]. Similarly, persistent changes in H3K9, increased dimethylation and reduced trimethylation, were observed in the NAc of adult rats following adolescent THC exposure [260]. Another study observed a significant increase of H3K9me2 in the hippocampus and the amygdala of female rats exposed to THC during adolescence [261]. Moreover, using the same adolescent THC exposure, there was an enhancement in H3K9me3 in the nucleus accumbens, hippocampus, and PFC [261,262]. Preconception THC exposure also disrupts DNA methylation in the NAc, with cross-generational effects. In a study comparing rats with or without parental THC exposure, 1027 differentially methylated regions (406 hypermethylated and 621 hypomethylated) associated with parental THC exposure were found in the subsequent generation, even though they were not directly exposed to the drug [263].

Confirming the correlation between the inheritance of paternal epigenetic changes and cannabinoid exposure, developmental changes in the offspring were associated with premating paternal THC exposure [264]. Exposure to cannabinoids has also been associated with changes in sperm DNA methylation. The analysis of sperm DNA from adult rats exposed to 2 mg/kg of THC for 12 days identified 627 genes whose methylation status was altered [265]. Similarly, significant differential methylation of genes related to neurodevelopment was observed in the sperm of rats exposed to THC via oral gavage [266]. Similarly to the preclinical reports, substantial changes in both hypo- and hyper-DNA methylation, with the latter predominating, were determined in the sperm methylome of marijuana smokers [265]. The impact of cannabis exposure on DNA methylation status also was investigated directly in human spermatogenesis *in vitro*. The results revealed alterations in DNA methylation levels of genes related to autism, HCN1, and NR4A2 [267]. These studies provide compelling evidence that preconception exposure to cannabinoids can impact reproduction and paternal epigenetic inheritance, potentially leading to altered DNA methylation patterns that have an impact on gene expression and developmental outcomes in offspring.

Altogether these findings support the idea that the eCB system is involved in regulating epigenetic mechanisms and has an essential role in the effects of addictive drugs. Since this response profile also was observed with stress exposure and considering the role of stress in the neurobiology of the substance use disorder, future studies might evaluate the involvement of the eCB system in the modulation of drug addiction by stress.

## Final remarks

In the last two decades, much attention was directed toward understanding how exposure to different stressors could result in long-term changes in the organism that could result in psychiatric disorders. In this context, a boom of studies evaluating epigenetic changes in animal models and a run to find epigenetic markers related to psychiatric conditions arose, bringing many new understandings in the neurobiology of psychiatric conditions.

Among several physiological systems affected by epigenetic modulation, one has, in particular, been in the spotlight of scientists for more than 20 years: the endocannabinoid system. As overviewed in this review, the eCB system has a fundamental role in controlling many functions, including the fine control of stress response and circuits involved in drug abuse. Although not fully explored, eCB system genes are sensitive to epigenetic control [183,184,268,269]. The discussed evidence highlights the importance of epigenetic mechanisms in the eCB system response to stress, drugs of abuse, and the dysfunctions caused by them. As epigenetic marks can persist, the long-term alteration in the expression of cannabinoid-related proteins may be part of triggering diseases, particularly after stressors or substance use disorder. More recently, as discussed, many studies have investigated if the behavioral consequences of exposure to stressors in animals’ models could result in epigenetic regulation of the eCB system. Changes in miRNAs that regulate eCB system molecules, for example, are observed after acute protocols of stress in animal models but also in postmortem brains of depressive subjects. Epigenetic changes can persist through generations, indicating how stress and drug exposure, for example, can modify the neurobiology along the generations.

As also discussed, in animal models, cannabinoids, including THC and CBD, promote several behavioral changes related to psychiatric disorders and induce epigenetic modifications, mainly related to DNA methylation and histone modifications. Besides, *Cannabis* use in humans appears to induce epigenetic changes not only in the eCB system but also in the dopaminergic system and others, indicating a potential mechanism by which it could lead to psychiatric disorders, including substance use disorder. Finally, exposure to cannabinoids during critical periods of brain development can induce persistent brain and behavioral changes in adulthood.

**Figure 2:**
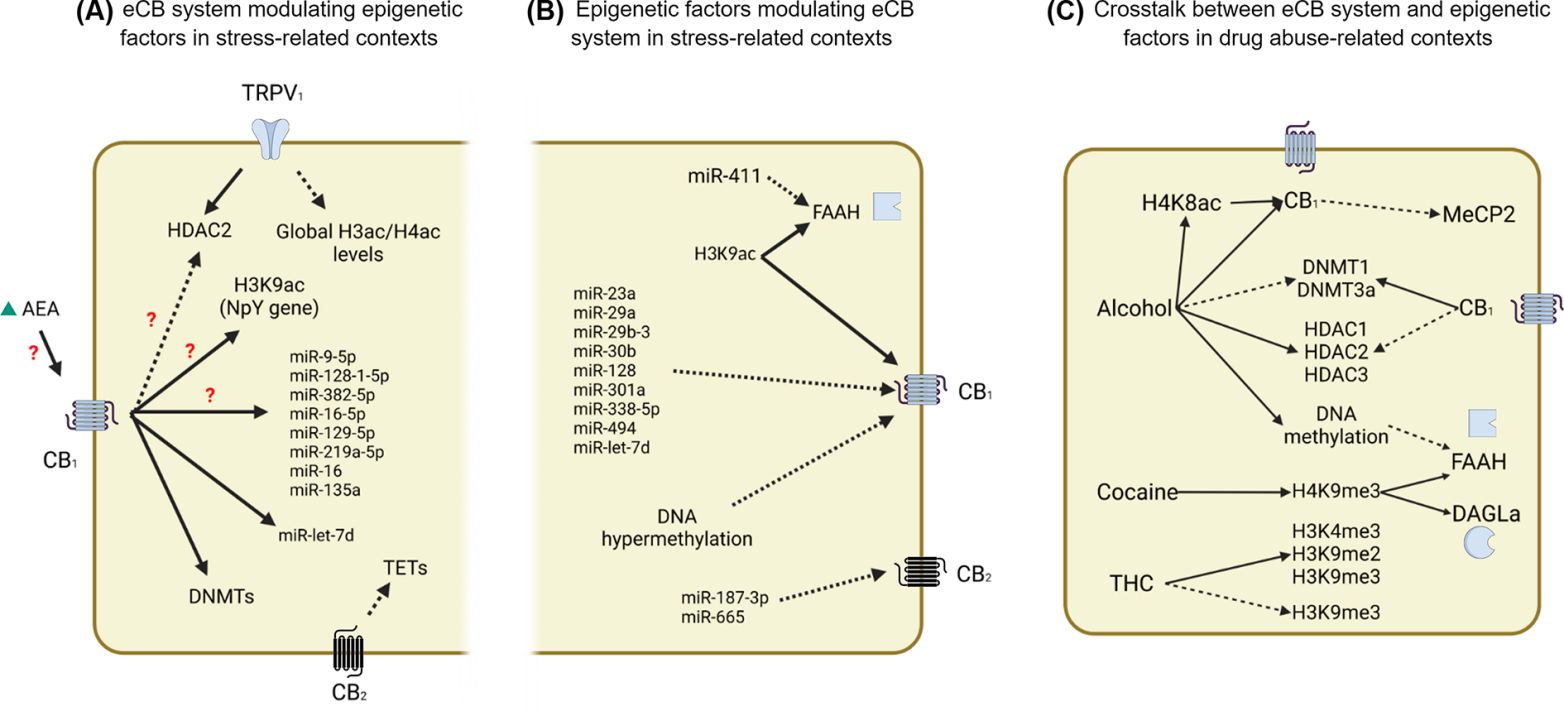
Mechanisms involved in the cross-talk between the eCB system and epigenetic mechanisms in stress- and drug abuse-related contexts (**A**) In stressful contexts, interference with the eCB system can modulate a wide range of epigenetic factors. CB_1_ receptors, for example, modulate the expression of DNMTs and the microRNA let-7d. Moreover, the inhibition of FAAH and consequent increase in AEA levels, which may act at CB_1_ receptors, increases the expression of several miRNAs (miR-9-5p, miR-128-1-5p, miR-382-5p, miR-16-5p, miR-129-5p, miR-219a-5p, miR-16, and miR-135a) and H3K9ac levels at npy gene, and decreases the expression and activity of HDAC2. Moreover, TRPV_1_ receptors activation increases the expression of HDAC2 and reduces global H3/H4 acetylation levels. Finally, CB_2_ receptors have been shown to reduce TET enzyme levels. (**B**) Stress can regulate eCB genes through epigenetics tools. CB1 expression is reported to be sensitive to hypermethylation and increased levels of H3K9ac of the gene and affected by miRNAs (miR-23a, miR-29a, miR-29b-3, miR-30b, miR-128, miR-301a, miR-338-5p, miR-494, and miR-let-7d). FAAH expression may also be altered by H3K9ac and the miR-411. Furthermore, CB2 is one target of miR-187-3p and miR-665 expression. (**C**) The cross-talk between the systems in the context of drug abuse is very diversified since drugs with different mechanisms of action promote different alterations. For example, alcohol increases H4K8ac in the CB1 gene, and its protein expression is related to the down-regulation of MeCP2. Alcohol also down-regulates DNMT1 and DNMT3a and upregulates HDAC1, HDAC2, and HDAC3, and all these effects are blocked by CB1 antagonism. Moreover, DNA methylation of the FAAH gene is affected by alcohol exposure. Cocaine consumption is reported to increase H4K9me3 in FAAH and DAGLα genes. Regarding exposure to cannabinoids, THC induces global levels of H3K4me3 and H3K9me2 and can increase or decrease H3K9me3 depending on the exposure. More details about these mechanisms can be found in the main text. Dashed arrows indicate inhibition or reduction. Continuous arrow indicate induction or increase. Question mark indicates that the CB1 involvement after FAAH inhibition was not directed tested.

As summarized in this review, therefore, there appears to have a close relationship between modulation of the eCB system and evaluation of epigenetic changes (DNA methylation, histones modifications, and miRNAs) under stress conditions (Figure 2A) and how epigenetic markers under stress conditions, mainly miRNAs, influence the expression of eCB-related molecules (Figure 2B). Furthermore, common drugs of abuse, including alcohol, cocaine, and cannabis (THC), could promote their long-term effects by promoting epigenetic changes that impact the eCB system (Figure 2C). The elucidation of epigenetic mechanisms controlling, or being controlled by, the eCB system in stress-related disorders is essential to better understand the neurobiology of those disorders and to provide new treatment approaches. Finally, understanding the cross-talk between those systems can potentially lead to the identification of biomarkers, such as miRNAs, which could help to predict the course of the disease and treatment response.

## Data Availability

Data sharing is not applicable.

## Open Access

Open access for the present article was enabled after accepting the invitation to contribute with an article in the Neuronal Signaling journal, agreement with Portland Press and the Biochemical Society.

## Abbreviations

AID-APOBEC: activation-induced cytidine deaminase/apolipoprotein B mRNA-editing enzyme complex
ARS: acute restraint stress
AEA: anandamide
BLA: basolateral amygdala
CA: Ammon’s horn/Cornus Ammonics subfield
CB: cannabinoid receptor
CeA: central amygdala
CNS: central nervous system
CRS: chronic restraint stress
CSD: chronic social defeat
CUMS: chronic unpredictable mild stress
D2: dopamine receptor D2
DG: dentate gyrus
DAGL: diacylglycerol lipase
DNMT: DNA methyltransferase
eCB: endocannabinoid
FAAH: fatty acid amide hydrolase
GABA: gamma-aminobutyric acid
GC: glucocorticoid
GPR55: G protein-coupled receptor 55
GR: glucocorticoid receptor
HAT: histone acetyltransferase
HDAC: histone deacetylase
HPA: hypothalamus-pituitary-adrenal
IL: infralimbic
MAGL: monoacylglycerol lipase
MBP: methyl-CpG binding protein
MDD: major depressive disease
MeCP2: methyl CpG binding protein 2
MeA: medial amygdala
miRNA: micro RNA
mPFC: medial prefrontal cortex
MS: maternal separation
NAc: nucleus accumbens
NAPE-PLD: N-acyl phosphatidylethanolamine phospholipase D
NpY: neuropeptide Y
NR3C1: glucocorticoid receptor gene
PBMC: peripheral blood mononuclear cell
PD7: postnatal day 7
PFC: prefrontal cortex
PKA: protein kinase A
PL: prelimbic
PPARγ: peroxisome proliferator-activated receptor gamma
PTSD: post-traumatic stress disorder
SA: self-administration
SLC6A4: solute carrier family 6 member 4
TET: ten-eleven translocation enzyme
TRPV1: vanilloid receptor 1
2-AG: 2-arachidonoylglycerol
5mC: cytosine methylated at the 5′ position

## References

[1] Godoy L.D., Rossignoli M.T., Delfino-Pereira P., Garcia-Cairasco N. and de Lima Umeoka E.H. (2018) A comprehensive overview on stress neurobiology: basic concepts and clinical implications. Front Behav. Neurosci. 12 1–23 10.3389/fnbeh.2018.00127 30034327 PMC6043787

[2] Yamamoto K.R. (1985) Steroid receptor regulated transcription of specific genes and gene networks. Annu. Rev. Genet. 19, 209–252 10.1146/annurev.ge.19.120185.001233 3909942

[3] Wang J.-C., Derynck M.K., Nonaka D.F., Khodabakhsh D.B., Haqq C. and Yamamoto K.R. (2004) Chromatin immunoprecipitation (ChIP) scanning identifies primary glucocorticoid receptor target genes. Proc. Natl. Acad. Sci. 101, 15603–15608 10.1073/pnas.0407008101 15501915 PMC524211

[4] Murphy M.D. and Heller E.A. (2022) Convergent actions of stress and stimulants via epigenetic regulation of neural circuitry. Trends Neurosci. 45, 955–967 10.1016/j.tins.2022.10.001 36280459 PMC9671852

[5] Zhang W.-H., Zhang J.-Y., Holmes A. and Pan B.-X. (2021) Amygdala circuit substrates for stress adaptation and adversity. Biol. Psychiatry 89, 847–856 10.1016/j.biopsych.2020.12.026 33691931

[6] Alexandra Kredlow M., Fenster R.J., Laurent E.S., Ressler K.J. and Phelps E.A. (2022) Prefrontal cortex, amygdala, and threat processing: implications for PTSD. Neuropsychopharmacology 47, 247–259 10.1038/s41386-021-01155-7 34545196 PMC8617299

[7] Sousa N. (2016) The dynamics of the stress neuromatrix. Mol. Psychiatry 21, 302–312 10.1038/mp.2015.196 26754952 PMC4759204

[8] Le Merre P., Ährlund-Richter S. and Carlén M. (2021) The mouse prefrontal cortex: Unity in diversity. Neuron 109, 1925–1944 10.1016/j.neuron.2021.03.035 33894133

[9] Friedman N.P. and Robbins T.W. (2022) The role of prefrontal cortex in cognitive control and executive function. Neuropsychopharmacology 47, 72–89 10.1038/s41386-021-01132-0 34408280 PMC8617292

[10] McKlveen J.M., Myers B. and Herman J.P. (2015) The medial prefrontal cortex: coordinator of autonomic, neuroendocrine and behavioural responses to stress. J. Neuroendocrinol. 27, 446–456 10.1111/jne.12272 25737097 PMC4580281

[11] Manoocheri K. and Carter A.G. (2022) Rostral and caudal basolateral amygdala engage distinct circuits in the prelimbic and infralimbic prefrontal cortex. eLife 11, e82688 10.7554/eLife.82688 36476757 PMC9803354

[12] van Aerde K.I., Heistek T.S. and Mansvelder H.D. (2008) Prelimbic and infralimbic prefrontal cortex interact during fast network oscillations. PloS ONE 3, e2725 10.1371/journal.pone.0002725 18628964 PMC2444037

[13] Gabbott P.L.A., Warner T.A., Jays P.R.L., Salway P. and Busby S.J. (2005) Prefrontal cortex in the rat: projections to subcortical autonomic, motor, and limbic centers. J. Comp. Neurol. 492, 145–177 10.1002/cne.20738 16196030

[14] Vertes R.P. (2004) Differential projections of the infralimbic and prelimbic cortex in the rat. Synapse 51, 32–58 10.1002/syn.10279 14579424

[15] Popoli M., Yan Z., McEwen B.S. and Sanacora G. (2012) The stressed synapse: the impact of stress and glucocorticoids on glutamate transmission. Nat. Rev. Neurosci. 13, 22–37 10.1038/nrn3138 PMC3645314 22127301

[16] Gądek-Michalska A., Tadeusz J., Rachwalska P. and Bugajski J. (2013) Cytokines, prostaglandins and nitric oxide in the regulation of stress-response systems. Pharmacological Rep. 65, 1655–1662 10.1016/S1734-1140(13)71527-5 24553014

[17] Pandey G.N., Rizavi H.S., Ren X., Fareed J., Hoppensteadt D.A., Roberts R.C. et al. (2012) Proinflammatory cytokines in the prefrontal cortex of teenage suicide victims. J. Psychiatr. Res. 46, 57–63 10.1016/j.jpsychires.2011.08.006 21906753 PMC3224201

[18] Banasr M., Dwyer J.M. and Duman R.S. (2011) Cell atrophy and loss in depression: reversal by antidepressant treatment. Curr. Opin. Cell Biol. 23, 730–737 10.1016/j.ceb.2011.09.002 21996102 PMC3259683

[19] Rauch S.L., Shin L.M., Segal E., Pitman R.K., Carson M.A., McMullin K. et al. (2003) Selectively reduced regional cortical volumes in post-traumatic stress disorder. Neuroreport 14, 913–916 10.1097/01.wnr.0000071767.24455.10 12802174

[20] Arnsten A.F.T. (2015) Stress weakens prefrontal networks: molecular insults to higher cognition. Nat. Neurosci. 18, 1376–1385 10.1038/nn.4087 26404712 PMC4816215

[21] Woo E., Sansing L.H., Arnsten A.F.T. and Datta D. (2021) Chronic stress weakens connectivity in the prefrontal cortex: architectural and molecular changes. Chronic Stress 5, 24705470211029254 10.1177/24705470211029254 34485797 PMC8408896

[22] Sun Y., Gooch H. and Sah P. (2020) Fear conditioning and the basolateral amygdala. F1000Research 9, 53 10.12688/f1000research.21201.1 PMC6993823 32047613

[23] Duvarci S., Popa D. and Pare D. (2011) Central amygdala activity during fear conditioning. J. Neurosci. 31, 289–294 10.1523/JNEUROSCI.4985-10.2011 21209214 PMC3080118

[24] Hu F., Liang W., Zhang L., Wang H., Li Z. and Zhou Y. (2022) Hyperactivity of basolateral amygdala mediates behavioral deficits in mice following exposure to bisphenol A and its analogue alternative. Chemosphere 287, 132044 10.1016/j.chemosphere.2021.132044 34474391

[25] Inagaki R., Moriguchi S. and Fukunaga K. (2018) Aberrant amygdala-dependent fear memory in corticosterone-treated mice. Neuroscience 388, 448–459 10.1016/j.neuroscience.2018.08.004 30118751

[26] Siegle G.J., Steinhauer S.R., Thase M.E., Stenger V.A. and Carter C.S. (2002) Can't shake that feeling: event-related fMRI assessment of sustained amygdala activity in response to emotional information in depressed individuals. Biol. Psychiatry 51, 693–707 10.1016/S0006-3223(02)01314-8 11983183

[27] Lupien S.J., Parent S., Evans A.C., Tremblay R.E., Zelazo P.D., Corbo V. et al. (2011) Larger amygdala but no change in hippocampal volume in 10-year-old children exposed to maternal depressive symptomatology since birth. Proc. Natl. Acad. Sci. 108, 14324–14329 10.1073/pnas.1105371108 21844357 PMC3161565

[28] Frodl T., Meisenzahl E., Zetzsche T., Bottlender R., Born C., Groll C. et al. (2002) Enlargement of the amygdala in patients with a first episode of major depression. Biol. Psychiatry 51, 708–714 10.1016/S0006-3223(01)01359-2 11983184

[29] Dejean C., Courtin J., Rozeske R.R., Bonnet M.C., Dousset V., Michelet T. et al. (2015) Neuronal circuits for fear expression and recovery: recent advances and potential therapeutic strategies. Biol. Psychiatr. 78, 298–306 10.1016/j.biopsych.2015.03.017 25908496

[30] Thierry A.-M., Gioanni Y., Dégénétais E. and Glowinski J. (2000) Hippocampo-prefrontal cortex pathway: Anatomical and electrophysiological characteristics. Hippocampus 10, 411–419 10.1002/1098-1063(2000)10:4<411::AID-HIPO7>3.0.CO;2-A 10985280

[31] Ishikawa A. and Nakamura S. (2003) Convergence and interaction of hippocampal and amygdalar projections within the prefrontal cortex in the rat. J. Neurosci. 23, 9987–9995 10.1523/JNEUROSCI.23-31-09987.2003 14602812 PMC6740854

[32] Kim W.B. and Cho J.-H. (2017) Synaptic targeting of double-projecting ventral CA1 hippocampal neurons to the medial prefrontal cortex and basal amygdala. J. Neurosci. 37, 4868–4882 10.1523/JNEUROSCI.3579-16.2017 28385873 PMC6596479

[33] Çalışkan G. and Stork O. (2019) Hippocampal network oscillations at the interplay between innate anxiety and learned fear. Psychopharmacology (Berl.) 236, 321–338 10.1007/s00213-018-5109-z 30417233

[34] Asok A., Kandel E.R. and Rayman J.B. (2019) The neurobiology of fear generalization. Front Behav Neurosci. 12, 1–15 10.3389/fnbeh.2018.00329 30697153 PMC6340999

[35] Kim E.J., Pellman B. and Kim J.J. (2015) Stress effects on the hippocampus: a critical review. Learn. Memory 22, 411–416 10.1101/lm.037291.114 26286651 PMC4561403

[36] Webler R.D., Berg H., Fhong K., Tuominen L., Holt D.J., Morey R.A. et al. (2021) The neurobiology of human fear generalization: meta-analysis and working neural model. Neurosci. Biobehavioral Rev. 128, 421–436 10.1016/j.neubiorev.2021.06.035 34242718

[37] Murray F., Smith D.W. and Hutson P.H. (2008) Chronic low dose corticosterone exposure decreased hippocampal cell proliferation, volume and induced anxiety and depression like behaviours in mice. Eur. J. Pharmacol. 583, 115–127 10.1016/j.ejphar.2008.01.014 18289522

[38] Gurvits T.V., Shenton M.E., Hokama H., Ohta H., Lasko N.B., Gilbertson M.W. et al. (1996) Magnetic resonance imaging study of hippocampal volume in chronic, combat-related posttraumatic stress disorder. Biol. Psychiatry 40, 1091–1099 10.1016/S0006-3223(96)00229-6 8931911 PMC2910907

[39] Nolan M., Roman E., Nasa A., Levins K.J., O'Hanlon E., O'Keane V. et al. (2020) Hippocampal and amygdalar volume changes in major depressive disorder: a targeted review and focus on stress. Chronic Stress 4, 2470547020944553 10.1177/2470547020944553 33015518 PMC7513405

[40] Karl A., Schaefer M., Malta L.S., Dörfel D., Rohleder N. and Werner A. (2006) A meta-analysis of structural brain abnormalities in PTSD. Neurosci. Biobehav. Rev. 30, 1004–1031 10.1016/j.neubiorev.2006.03.004 16730374

[41] Bremner J.D., Randall P., Scott T.M., Bronen R.A., Seibyl J.P., Southwick S.M. et al. (1995) MRI-based measurement of hippocampal volume in patients with combat-related posttraumatic stress disorder. Am. J. Psychiatry 152, 973–981 10.1176/ajp.152.7.973 7793467 PMC3233767

[42] Gilbertson M.W., Shenton M.E., Ciszewski A., Kasai K., Lasko N.B., Orr S.P. et al. (2002) Smaller hippocampal volume predicts pathologic vulnerability to psychological trauma. Nat. Neurosci. 5, 1242–1247 10.1038/nn958 12379862 PMC2819093

[43] Cristino L., Bisogno T. and Di Marzo V. (2020) Cannabinoids and the expanded endocannabinoid system in neurological disorders. Nat. Rev. Neurol. 16, 9–29 10.1038/s41582-019-0284-z 31831863

[44] Witkin J.M., Tzavara E.T. and Nomikos G.G. (2005) A role for cannabinoid CB1 receptors in mood and anxiety disorders. Behav. Pharmacol. 16, 333–352 10.1097/00008877-200509000-00005 16148437

[45] Marsicano G., Wotjak C.T., Azad S.C., Bisogno T., Rammes G., Cascio M.G. et al. (2002) The endogenous cannabinoid system controls extinction of aversive memories. Nature 418, 530–534 10.1038/nature00839 12152079

[46] Lisboa S.F., Gomes F.V., Silva A.L., Uliana D.L., Camargo L.H.A., Guimarães F.S. et al. (2015) Increased contextual fear conditioning in iNOS knockout mice: additional evidence for the involvement of nitric oxide in stress-related disorders and contribution of the endocannabinoid system. Int. J. Neuropsychopharmacolog. 18, pyv005 10.1093/ijnp/pyv005 PMC4571624 25618404

[47] Terzian A.L.B., Dos Reis D.G., Guimarães F.S., Corrêa F.M.A. and Resstel L.B.M. (2014) Medial prefrontal cortex transient receptor potential vanilloid type 1 (TRPV1) in the expression of contextual fear conditioning in Wistar rats. Psychopharmacology (Berl.) 231, 149–157 10.1007/s00213-013-3211-9 23922023

[48] Gobira P.H., Lima I.V., Batista L.A., de Oliveira A.C., Resstel L.B., Wotjak C.T. et al. (2017) N-arachidonoyl-serotonin, a dual FAAH and TRPV1 blocker, inhibits the retrieval of contextual fear memory: Role of the cannabinoid CB1 receptor in the dorsal hippocampus. J. Psychopharmacol. 31, 750–756 10.1177/0269881117691567 28583049

[49] Marzo V.D., Bifulco M. and Petrocellis L.D. (2004) The endocannabinoid system and its therapeutic exploitation. Nat. Rev. Drug Discovery 3, 771–784 10.1038/nrd1495 15340387

[50] Bassir Nia A., Bender R. and Harpaz-Rotem I. (2019) Endocannabinoid system alterations in posttraumatic stress disorder: a review of developmental and accumulative effects of trauma. Chronic Stress 3, 2470547019864096 10.1177/2470547019864096 31660473 PMC6816276

[51] Patel S., Hill M.N., Cheer J.F., Wotjak C.T. and Holmes A. (2017) The endocannabinoid system as a target for novel anxiolytic drugs. Neurosci. Biobehavioral Rev. 76, 56–66 10.1016/j.neubiorev.2016.12.033 PMC5407316 28434588

[52] Hill M.N., Miller G.E., Carrier E.J., Gorzalka B.B. and Hillard C.J. (2009) Circulating endocannabinoids and N-acyl ethanolamines are differentially regulated in major depression and following exposure to social stress. Psychoneuroendocrinology 34, 1257–1262 10.1016/j.psyneuen.2009.03.013 19394765 PMC2716432

[53] Hungund B.L., Vinod K.Y., Kassir S.A., Basavarajappa B.S., Yalamanchili R., Cooper T.B. et al. (2004) Upregulation of CB1 receptors and agonist-stimulated [35S]GTPγS binding in the prefrontal cortex of depressed suicide victims. Mol. Psychiatry 9, 184–190 10.1038/sj.mp.4001376 14966476

[54] Gonda X., Petschner P., Eszlari N., Sutori S., Gal Z., Koncz S. et al. (2019) Effects of different stressors are modulated by different neurobiological systems: the role of GABA-A versus CB1 receptor gene variants in anxiety and depression. Front Cell Neurosci. 13, 1–12 10.3389/fncel.2019.00138 31024264 PMC6467241

[55] Mitjans M., Serretti A., Fabbri C., Gastó C., Catalán R., Fañanás L. et al. (2013) Screening genetic variability at the CNR1 gene in both major depression etiology and clinical response to citalopram treatment. Psychopharmacology (Berl.) 227, 509–519 10.1007/s00213-013-2995-y 23407780

[56] Domschke K., Dannlowski U., Ohrmann P., Lawford B., Bauer J., Kugel H. et al. (2008) Cannabinoid receptor 1 (CNR1) gene: Impact on antidepressant treatment response and emotion processing in Major Depression. Eur. Neuropsychopharmacol. 18, 751–759 10.1016/j.euroneuro.2008.05.003 18579347

[57] Christensen R., Kristensen P.K., Bartels E.M., Bliddal H. and Astrup A. (2007) Efficacy and safety of the weight-loss drug rimonabant: a meta-analysis of randomised trials. Lancet North Am. Ed. 370, 1706–1713 10.1016/S0140-6736(07)61721-8 18022033

[58] Hill M.N., Ho W.-S.V., Hillard C.J. and Gorzalka B.B. (2008) Differential effects of the antidepressants tranylcypromine and fluoxetine on limbic cannabinoid receptor binding and endocannabinoid contents. J. Neural Transm. 115, 1673–1679 10.1007/s00702-008-0131-7 18974922 PMC2992975

[59] Neumeister A., Normandin M.D., Pietrzak R.H., Piomelli D., Zheng M.Q., Gujarro-Anton A. et al. (2013) Elevated brain cannabinoid CB1 receptor availability in post-traumatic stress disorder: a positron emission tomography study. Mol. Psychiatry 18, 1034–1040 10.1038/mp.2013.61 23670490 PMC3752332

[60] Mayo L.M., Asratian A., Lindé J., Holm L., Nätt D., Augier G. et al. (2020) Protective effects of elevated anandamide on stress and fear-related behaviors: translational evidence from humans and mice. Mol. Psychiatry 25, 993–1005 10.1038/s41380-018-0215-1 30120421

[61] Zabik N.L., Iadipaolo A.S., Marusak H.A., Peters C., Burghardt K. and Rabinak C.A. (2022) A common genetic variant in fatty acid amide hydrolase is linked to alterations in fear extinction neural circuitry in a racially diverse, nonclinical sample of adults. J. Neurosci. Res. 100, 744–761 10.1002/jnr.24860 34051704 PMC8628026

[62] Lazary J., Eszlari N., Juhasz G. and Bagdy G. (2016) Genetically reduced FAAH activity may be a risk for the development of anxiety and depression in persons with repetitive childhood trauma. Eur. Neuropsychopharmacol. 26, 1020–1028 10.1016/j.euroneuro.2016.03.003 27005594

[63] Lazary J., Eszlari N., Juhasz G. and Bagdy G. (2019) A functional variant of CB2 receptor gene interacts with childhood trauma and FAAH gene on anxious and depressive phenotypes. J. Affect. Disord. 257, 716–722 10.1016/j.jad.2019.07.083 31382124

[64] Ney L.J., Crombie K.M., Mayo L.M., Felmingham K.L., Bowser T. and Matthews A. (2022) Translation of animal endocannabinoid models of PTSD mechanisms to humans: Where to next? Neurosci. Biobehavioral Rev. 132, 76–91 10.1016/j.neubiorev.2021.11.040 34838529

[65] Lisboa S.F., Vila-Verde C., Rosa J., Uliana D.L., Stern C.A.J., Bertoglio L.J. et al. (2019) Tempering aversive/traumatic memories with cannabinoids: a review of evidence from animal and human studies. Psychopharmacology (Berl.) 236, 201–226 10.1007/s00213-018-5127-x 30604182

[66] Ney L.J., Matthews A., Hsu C.-M.K., Zuj D.V., Nicholson E., Steward T. et al. (2021) Cannabinoid polymorphisms interact with plasma endocannabinoid levels to predict fear extinction learning. Depress. Anxiety 38, 1087–1099 10.1002/da.23170 34151472

[67] Navarrete F., García-Gutiérrez M.S., Jurado-Barba R., Rubio G., Gasparyan A., Austrich-Olivares A. et al. (2020) Endocannabinoid system components as potential biomarkers in psychiatry. Front Psychiatry 11, 1–30 10.3389/fpsyt.2020.00315 32395111 PMC7197485

[68] Mayo L.M., Rabinak C.A., Hill M.N. and Heilig M. (2022) Targeting the endocannabinoid system in the treatment of posttraumatic stress disorder: a promising case of preclinical-clinical translation? Biol. Psychiatry 91, 262–272 10.1016/j.biopsych.2021.07.019 34598785 PMC11097652

[69] Haller J., Bakos N., Szirmay M., Ledent C. and Freund T.F. (2002) The effects of genetic and pharmacological blockade of the CB1 cannabinoid receptor on anxiety. Eur. J. Neurosci. 16, 1395–1398 10.1046/j.1460-9568.2002.02192.x 12405999

[70] Aso E., Ozaita A., Serra M.-À. and Maldonado R. (2011) Genes differentially expressed in CB1 knockout mice: Involvement in the depressive-like phenotype. Eur. Neuropsychopharmacol. 21, 11–22 10.1016/j.euroneuro.2010.06.007 20692131

[71] Valverde O. and Torrens M. (2012) CB1 receptor-deficient mice as a model for depression. Neuroscience 204, 193–206 10.1016/j.neuroscience.2011.09.031 21964469

[72] Martin M., Ledent C., Parmentier M., Maldonado R. and Valverde O. (2002) Involvement of CB1 cannabinoid receptors in emotional behaviour. Psychopharmacology (Berl.) 159, 379–387 10.1007/s00213-001-0946-5 11823890

[73] Mikics É., Dombi T., Barsvári B., Varga B., Ledent C., Freund T.F. et al. (2006) The effects of cannabinoids on contextual conditioned fear in CB1 knockout and CD1 mice. Behav. Pharmacol. 17, 10.1097/00008877-200605000-00003 16572000

[74] Beyer C.E., Dwyer J.M., Piesla M.J., Platt B.J., Shen R., Rahman Z. et al. (2010) Depression-like phenotype following chronic CB1 receptor antagonism. Neurobiol. Dis. 39, 148–155 10.1016/j.nbd.2010.03.020 20381618

[75] O'Brien L.D., Wills K.L., Segsworth B., Dashney B., Rock E.M., Limebeer C.L. et al. (2013) Effect of chronic exposure to rimonabant and phytocannabinoids on anxiety-like behavior and saccharin palatability. Pharmacol. Biochem. Behav. 103, 597–602 10.1016/j.pbb.2012.10.008 23103902

[76] Rutkowska M. and Jachimczuk O. (2004) Antidepressant–like properties of ACEA (arachidonyl-2-chloroethylamide), the selective agonist of CB1 receptors. Acta Pol. Pharm. 61, 165–167 15493300

[77] Hill M.N. and Gorzalka B.B. (2005) Pharmacological enhancement of cannabinoid CB1 receptor activity elicits an antidepressant-like response in the rat forced swim test. Eur. Neuropsychopharmacol. 15, 593–599 10.1016/j.euroneuro.2005.03.003 15916883

[78] Haller J., Varga B., Ledent C. and Freund T.F. (2004) CB1 cannabinoid receptors mediate anxiolytic effects: convergent genetic and pharmacological evidence with CB1-specific agents. Behav. Pharmacol. 15, 299–304 10.1097/01.fbp.0000135704.56422.40 15252281

[79] Ganon-Elazar E. and Akirav I. (2013) Cannabinoids and traumatic stress modulation of contextual fear extinction and GR expression in the amygdala-hippocampal-prefrontal circuit. Psychoneuroendocrinology 38, 1675–1687 10.1016/j.psyneuen.2013.01.014 23433741

[80] Laricchiuta D., Centonze D. and Petrosini L. (2013) Effects of endocannabinoid and endovanilloid systems on aversive memory extinction. Behav. Brain Res. 256, 101–107 10.1016/j.bbr.2013.08.010 23948212

[81] García-Gutiérrez M.S. and Manzanares J. (2010) Overexpression of CB2 cannabinoid receptors decreased vulnerability to anxiety and impaired anxiolytic action of alprazolam in mice. J. Psychopharmacol. 25, 111–120 10.1177/0269881110379507 20837564

[82] García-Gutiérrez M.S., García-Bueno B., Zoppi S., Leza J.C. and Manzanares J. (2012) Chronic blockade of cannabinoid CB2 receptors induces anxiolytic-like actions associated with alterations in GABA(A) receptors. Br. J. Pharmacol. 165, 951–964 10.1111/j.1476-5381.2011.01625.x 21838753 PMC3312491

[83] García-Gutiérrez M.S., Pérez-Ortiz J.M., Gutiérrez-Adán A. and Manzanares J. (2010) Depression-resistant endophenotype in mice overexpressing cannabinoid CB(2) receptors. Br. J. Pharmacol. 160, 1773–1784 10.1111/j.1476-5381.2010.00819.x 20649579 PMC2936848

[84] Li Y. and Kim J. (2016) CB2 cannabinoid receptor knockout in mice impairs contextual long-term memory and enhances spatial working memory. Neural Plast. 2016, 9817089 10.1155/2016/9817089 26819779 PMC4706977

[85] Gerdeman G. and Lovinger D.M. (2001) CB1 cannabinoid receptor inhibits synaptic release of glutamate in rat dorsolateral striatum. J. Neurophysiol. 85, 468–471 10.1152/jn.2001.85.1.468 11152748

[86] Polissidis A., Galanopoulos A., Naxakis G., Papahatjis D., Papadopoulou-Daifoti Z. and Antoniou K. (2013) The cannabinoid CB1 receptor biphasically modulates motor activity and regulates dopamine and glutamate release region dependently. Int. J. Neuropsychopharmacolog. 16, 393–403 10.1017/S1461145712000156 22391102

[87] Ferreira S.G., Teixeira F.M., Garção P., Agostinho P., Ledent C., Cortes L. et al. (2012) Presynaptic CB1 cannabinoid receptors control frontocortical serotonin and glutamate release – Species differences. Neurochem. Int. 61, 219–226 10.1016/j.neuint.2012.05.009 22609378 PMC3408788

[88] Blázquez C., Chiarlone A., Bellocchio L., Resel E., Pruunsild P., García-Rincón D. et al. (2015) The CB1 cannabinoid receptor signals striatal neuroprotection via a PI3K/Akt/mTORC1/BDNF pathway. Cell Death Differentiation 22, 1618–1629 10.1038/cdd.2015.11 25698444 PMC4563779

[89] Aso E., Ozaita A., Valdizán E.M., Ledent C., Pazos Á., Maldonado R. et al. (2008) BDNF impairment in the hippocampus is related to enhanced despair behavior in CB1 knockout mice. J. Neurochem. 105, 565–572 10.1111/j.1471-4159.2007.05149.x 18047561

[90] Zoppi S., Madrigal J.L., Caso J.R., García-Gutiérrez M.S., Manzanares J., Leza J.C. et al. (2014) Regulatory role of the cannabinoid CB2receptor in stress-induced neuroinflammation in mice. Br. J. Pharmacol. 171, 2814–2826 10.1111/bph.12607 24467609 PMC4243857

[91] Lisboa S.F., Gomes F.V., Guimaraes F.S. and Campos A.C. (2016) Microglial cells as a link between cannabinoids and the immune hypothesis of psychiatric disorders. Front Neurol. 7, 10.3389/fneur.2016.00005 PMC4729885 26858686

[92] Lisboa S.F., Niraula A., Resstel L.B., Guimaraes F.S., Godbout J.P. and Sheridan J.F. (2018) Repeated social defeat-induced neuroinflammation, anxiety-like behavior and resistance to fear extinction were attenuated by the cannabinoid receptor agonist WIN55,212-2. Neuropsychopharmacology 43, 1924–1933 10.1038/s41386-018-0064-2 29786066 PMC6046035

[93] Coelho A.A., Vila-Verde C., Sartim A.G., Uliana D.L., Braga L.A., Guimarães F.S. et al. (2022) Inducible nitric oxide synthase inhibition in the medial prefrontal cortex attenuates the anxiogenic-like effect of acute restraint stress via CB1 receptors. Front Psychiatry 13, 1–11 10.3389/fpsyt.2022.923177 PMC9330908 35911236

[94] Hill M.N., McLaughlin R.J., Pan B., Fitzgerald M.L., Roberts C.J., Lee T.T.Y. et al. (2011) Recruitment of prefrontal cortical endocannabinoid signaling by glucocorticoids contributes to termination of the stress response. J. Neurosci. 31, 10506–10515 10.1523/JNEUROSCI.0496-11.2011 21775596 PMC3179266

[95] Lyko F. (2018) The DNA methyltransferase family: a versatile toolkit for epigenetic regulation. Nat. Rev. Genet. 19, 81–92 10.1038/nrg.2017.80 29033456

[96] Chin E.W.M. and Goh E.L.K. (2019) MeCP2 dysfunction in rett syndrome and neuropsychiatric disorders. Methods Mol. Biol. 2011, 573–591 10.1007/978-1-4939-9554-7_33 31273722

[97] Du Q., Luu P.L., Stirzaker C. and Clark S.J. (2015) Methyl-CpG-binding domain proteins: readers of the epigenome. Epigenomics 7, 1051–1073 10.2217/epi.15.39 25927341

[98] Ehrlich M. (2019) DNA hypermethylation in disease: mechanisms and clinical relevance. Epigenetics 14, 1141–1163 10.1080/15592294.2019.1638701 31284823 PMC6791695

[99] Vinkers C.H., Kalafateli A.L., Rutten B.P., Kas M.J., Kaminsky Z., Turner J.D. et al. (2015) Traumatic stress and human DNA methylation: a critical review. Epigenomics 7, 593–608 10.2217/epi.15.11 26111031

[100] Klengel T., Pape J., Binder E.B. and Mehta D. (2014) The role of DNA methylation in stress-related psychiatric disorders. Neuropharmacology 80, 115–132 10.1016/j.neuropharm.2014.01.013 24452011

[101] Zhang Y. and Liu C. (2022) Evaluating the challenges and reproducibility of studies investigating DNA methylation signatures of psychological stress. Epigenomics 14, 405–421 10.2217/epi-2021-0190 35170363 PMC8978984

[102] Bakusic J., Schaufeli W., Claes S. and Godderis L. (2017) Stress, burnout and depression: A systematic review on DNA methylation mechanisms. J. Psychosom. Res. 92, 34–44 10.1016/j.jpsychores.2016.11.005 27998510

[103] Argentieri M.A., Nagarajan S., Seddighzadeh B., Baccarelli A.A. and Shields A.E. (2017) Epigenetic pathways in human disease: the impact of DNA methylation on stress-related pathogenesis and current challenges in biomarker development. EBioMedicine 18, 327–350 10.1016/j.ebiom.2017.03.044 28434943 PMC5405197

[104] Steiger H., Labonté B., Groleau P., Turecki G. and Israel M. (2013) Methylation of the glucocorticoid receptor gene promoter in bulimic women: Associations with borderline personality disorder, suicidality, and exposure to childhood abuse. Int. J. Eat. Disord. 46, 246–255 10.1002/eat.22113 23417893

[105] Labonté B., Suderman M., Maussion G., Navaro L., Yerko V., Mahar I. et al. (2012) Genome-wide epigenetic regulation by early-life trauma. Arch. Gen. Psychiatry 69, 722–731 10.1001/archgenpsychiatry.2011.2287 22752237 PMC4991944

[106] Perroud N., Paoloni-Giacobino A., Prada P., Olié E., Salzmann A., Nicastro R. et al. (2011) Increased methylation of glucocorticoid receptor gene (NR3C1) in adults with a history of childhood maltreatment: a link with the severity and type of trauma. Transl. Psychiatry 1, e59 10.1038/tp.2011.60 22832351 PMC3309499

[107] Radtke K.M., Ruf M., Gunter H.M., Dohrmann K., Schauer M., Meyer A. et al. (2011) Transgenerational impact of intimate partner violence on methylation in the promoter of the glucocorticoid receptor. Transl. Psychiatry 1, e21 10.1038/tp.2011.21 22832523 PMC3309516

[108] Tyrka A.R., Price L.H., Marsit C., Walters O.C. and Carpenter L.L. (2012) Childhood adversity and epigenetic modulation of the leukocyte glucocorticoid receptor: preliminary findings in healthy adults. PloS ONE 7, e30148 10.1371/journal.pone.0030148 22295073 PMC3266256

[109] Labonté B., Azoulay N., Yerko V., Turecki G. and Brunet A. (2014) Epigenetic modulation of glucocorticoid receptors in posttraumatic stress disorder. Transl. Psychiatry 4, e368 10.1038/tp.2014.3 24594779 PMC3966043

[110] Vukojevic V., Kolassa I.T., Fastenrath M., Gschwind L., Spalek K., Milnik A. et al. (2014) Epigenetic modification of the glucocorticoid receptor gene is linked to traumatic memory and post-traumatic stress disorder risk in genocide survivors. J. Neurosci. 34, 10274–10284 10.1523/JNEUROSCI.1526-14.2014 25080589 PMC6608273

[111] Na K.-S., Chang H.S., Won E., Han K.-M., Choi S., Tae W.S. et al. (2014) Association between glucocorticoid receptor methylation and hippocampal subfields in major depressive disorder. PLoS ONE 9, e85425 10.1371/journal.pone.0085425 24465557 PMC3897456

[112] Yehuda R., Flory J.D., Bierer L.M., Henn-Haase C., Lehrner A., Desarnaud F. et al. (2015) Lower methylation of glucocorticoid receptor gene promoter 1F in Peripheral blood of veterans with posttraumatic stress disorder. Biol. Psychiatry 77, 356–364 10.1016/j.biopsych.2014.02.006 24661442

[113] McGowan P.O., Sasaki A., D'Alessio A.C., Dymov S., Labonté B., Szyf M. et al. (2009) Epigenetic regulation of the glucocorticoid receptor in human brain associates with childhood abuse. Nat. Neurosci. 12, 342–348 10.1038/nn.2270 19234457 PMC2944040

[114] Suderman M., McGowan P.O., Sasaki A., Huang T.C.T., Hallett M.T., Meaney M.J. et al. (2012) Conserved epigenetic sensitivity to early life experience in the rat and human hippocampus. Proc. Natl. Acad. Sci. 109, 17266–17272 10.1073/pnas.1121260109 23045659 PMC3477392

[115] Beach S.R.H., Brody G.H., Todorov A.A., Gunter T.D. and Philibert R.A. (2010) Methylation at SLC6A4 is linked to family history of child abuse: An examination of the Iowa Adoptee sample. Am. J. Med. Genet. B Neuropsychiatr. Genet. 153B, 710–713 10.1002/ajmg.b.31028 19739105 PMC2909112

[116] Koenen K.C., Uddin M., Chang S.-C., Aiello A.E., Wildman D.E., Goldmann E. et al. (2011) SLC6A4 methylation modifies the effect of the number of traumatic events on risk for posttraumatic stress disorder. Depress. Anxiety 28, 639–647 10.1002/da.20825 21608084 PMC3145829

[117] Alasaari J.S., Lagus M., Ollila H.M., Toivola A., Kivimäki M., Vahtera J. et al. (2012) Environmental stress affects DNA methylation of a CpG rich promoter region of serotonin transporter gene in a nurse cohort. PLoS ONE 7, e45813 10.1371/journal.pone.0045813 23029256 PMC3461019

[118] Kang H.-J., Kim J.-M., Stewart R., Kim S.-Y., Bae K.-Y., Kim S.-W. et al. (2013) Association of SLC6A4 methylation with early adversity, characteristics and outcomes in depression. Prog. Neuropsychopharmacol. Biol. Psychiatry 44, 23–28 10.1016/j.pnpbp.2013.01.006 23333376

[119] Zhao J., Goldberg J., Bremner J.D. and Vaccarino V. (2013) Association between promoter methylation of serotonin transporter gene and depressive symptoms: A monozygotic twin study. Psychosom. Med. 75, 523–529 10.1097/PSY.0b013e3182924cf4 23766378 PMC3848698

[120] Wang D., Szyf M., Benkelfat C., Provençal N., Turecki G., Caramaschi D. et al. (2012) Peripheral SLC6A4 DNA methylation is associated with in vivo measures of human brain serotonin synthesis and childhood physical aggression. PloS ONE 7, e39501 10.1371/journal.pone.0039501 22745770 PMC3379993

[121] Kim J.M., Stewart R., Kang H.J., Kim S.W., Shin I.S., Kim H.R. et al. (2013) A longitudinal study of SLC6A4 DNA promoter methylation and poststroke depression. J. Psychiatr. Res. 47, 1222–1227 10.1016/j.jpsychires.2013.04.010 23702251

[122] Okada S., Morinobu S., Fuchikami M., Segawa M., Yokomaku K., Kataoka T. et al. (2014) The potential of SLC6A4 gene methylation analysis for the diagnosis and treatment of major depression. J. Psychiatr. Res. 53, 47–53 10.1016/j.jpsychires.2014.02.002 24657235

[123] Zhang Y., Sun Z., Jia J., Du T., Zhang N., Tang Y. et al. (2021) Overview of histone modification. In Histone Mutations and Cancer(Fang D. and Han J., eds), pp. 1–16, Springer Singapore, Singapore 10.1007/978-981-15-8104-5_1

[124] Lawrence M., Daujat S. and Schneider R. (2016) Lateral thinking: how histone modifications regulate gene expression. Trends Genet. 32, 42–56 10.1016/j.tig.2015.10.007 26704082

[125] Fiori L.M., Gross J.A. and Turecki G. (2012) Effects of histone modifications on increased expression of polyamine biosynthetic genes in suicide. Int. J. Neuropsychopharmacol. 15, 1161–1166 10.1017/S1461145711001520 22008221

[126] Golden S.A., Christoffel D.J., Heshmati M., Hodes G.E., Magida J., Davis K. et al. (2013) Epigenetic regulation of RAC1 induces synaptic remodeling in stress disorders and depression. Nat. Med. 19, 337–344 10.1038/nm.3090 23416703 PMC3594624

[127] Cruceanu C., Alda M., Nagy C., Freemantle E., Rouleau G.A. and Turecki G. (2013) H3K4 tri-methylation in synapsin genes leads to different expression patterns in bipolar disorder and major depression. Int. J. Neuropsychopharmacol. 16, 289–299 10.1017/S1461145712000363 22571925 PMC3564952

[128] Nestler E.J., Peña C.J., Kundakovic M., Mitchell A. and Akbarian S. (2016) Epigenetic basis of mental illness. Neuroscientist: a Rev. J. Bringing Neurobiol. Neurol. Psychiatry 22, 447–463 10.1177/1073858415608147 PMC4826318 26450593

[129] Thumfart K.M., Jawaid A., Bright K., Flachsmann M. and Mansuy I.M. (2022) Epigenetics of childhood trauma: Long term sequelae and potential for treatment. Neurosci. Biobehav. Rev. 132, 1049–1066 10.1016/j.neubiorev.2021.10.042 34742726

[130] Torres-Berrío A., Issler O., Parise E.M. and Nestler E.J. (2019) Unraveling the epigenetic landscape of depression: focus on early life stress. Dialogues Clin. Neurosci. 21, 341–357 31949402 10.31887/DCNS.2019.21.4/enestler PMC6952747

[131] Tsankova N.M., Berton O., Renthal W., Kumar A., Neve R.L. and Nestler E.J. (2006) Sustained hippocampal chromatin regulation in a mouse model of depression and antidepressant action. Nat. Neurosci. 9, 519–525 10.1038/nn1659 16501568

[132] Réus G.Z., Abelaira H.M., dos Santos M.A.B., Carlessi A.S., Tomaz D.B., Neotti M.V. et al. (2013) Ketamine and imipramine in the nucleus accumbens regulate histone deacetylation induced by maternal deprivation and are critical for associated behaviors. Behav. Brain Res. 256, 451–456 10.1016/j.bbr.2013.08.041 24004850

[133] Nghia N.A., Hirasawa T., Kasai H., Obata C., Moriishi K., Mochizuki K. et al. (2015) Long-term imipramine treatment increases N-methyl-d-aspartate receptor activity and expression via epigenetic mechanisms. Eur. J. Pharmacol. 752, 69–77 10.1016/j.ejphar.2015.02.010 25701723

[134] Schmauss C. (2015) An HDAC-dependent epigenetic mechanism that enhances the efficacy of the antidepressant drug fluoxetine. Sci. Rep. 5, 8171 10.1038/srep08171 25639887 PMC4313090

[135] Han A., Sung Y.B., Chung S.Y. and Kwon M.S. (2014) Possible additional antidepressant-like mechanism of sodium butyrate: targeting the hippocampus. Neuropharmacology 81, 292–302 10.1016/j.neuropharm.2014.02.017 24607816

[136] Ershadi A.S.B., Amini-Khoei H., Hosseini M.J. and Dehpour A.R. (2021) SAHA improves depressive symptoms, cognitive impairment and oxidative stress: rise of a new antidepressant class. Neurochem. Res. 46, 1252–1263 10.1007/s11064-021-03263-8 33576938

[137] Hsing C.H., Hung S.K., Chen Y.C., Wei T.S., Sun D.P., Wang J.J. et al. (2015) Histone deacetylase inhibitor trichostatin A ameliorated endotoxin-induced neuroinflammation and cognitive dysfunction. Mediators Inflamm. 2015, 163140 10.1155/2015/163140 26273133 PMC4530275

[138] Schroeder F.A., Lin C.L., Crusio W.E. and Akbarian S. (2007) Antidepressant-like effects of the histone deacetylase inhibitor, sodium butyrate, in the mouse. Biol. Psychiatry 62, 55–64 10.1016/j.biopsych.2006.06.036 16945350

[139] Panni S., Lovering R.C., Porras P. and Orchard S. (2020) Non-coding RNA regulatory networks. Biochim. Biophys. Acta. 1863, 194417 10.1016/j.bbagrm.2019.194417 31493559

[140] Lin R. and Turecki G. (2017) Noncoding RNAs in depression. In Neuroepigenomics in Aging and Disease(Delgado-Morales R., ed.), pp. 197–210, Springer International Publishing, Cham 10.1007/978-3-319-53889-1_11

[141] Snijders C., de Nijs L., Baker D.G., Hauger R.L., van den Hove D., Kenis G. et al. (2018) MicroRNAs in post-traumatic stress disorder. In Behavioral Neurobiology of PTSD(Vermetten E., Baker D.G. and Risbrough V.B., eds), pp. 23–46, Springer International Publishing, Cham 10.1007/7854_2017_32 29063484

[142] Murphy C.P. and Singewald N. (2019) Role of MicroRNAs in anxiety and anxiety-related disorders. In Behavioral Neurogenomics(Binder E.B. and Klengel T., eds), pp. 185–219, Springer International Publishing, Cham 10.1007/7854_2019_109 31485988

[143] O'Connor R.M., Gururajan A., Dinan T.G., Kenny P.J. and Cryan J.F. (2016) All roads lead to the miRNome: miRNAs have a central role in the molecular pathophysiology of psychiatric disorders. Trends Pharmacol. Sci. 37, 1029–1044 10.1016/j.tips.2016.10.004 27832923

[144] Dwivedi Y. (2018) MicroRNAs in depression and suicide: recent insights and future perspectives. J. Affect. Disord. 240, 146–154 10.1016/j.jad.2018.07.075 30071418 PMC6108934

[145] Hung Y.-Y., Wu M.-K., Tsai M.-C., Huang Y.-L. and Kang H.-Y. (2019) Aberrant expression of intracellular let-7e, miR-146a, and miR-155 correlates with severity of depression in patients with major depressive disorder and is ameliorated after antidepressant treatment. Cells 8, 1–12 10.3390/cells8070647 31252530 PMC6678487

[146] Bocchio-Chiavetto L., Maffioletti E., Bettinsoli P., Giovannini C., Bignotti S., Tardito D. et al. (2013) Blood microRNA changes in depressed patients during antidepressant treatment. Eur. Neuropsychopharmacol. 23, 602–611 10.1016/j.euroneuro.2012.06.013 22925464

[147] Baudry A., Mouillet-Richard S., Schneider B., Launay J.M. and Kellermann O. (2010) miR-16 targets the serotonin transporter: a new facet for adaptive responses to antidepressants. Science 329, 1537–1541 10.1126/science.1193692 20847275

[148] Wang S., Wu W. and Claret F.X. (2017) Mutual regulation of microRNAs and DNA methylation in human cancers. Epigenetics 12, 187–197 10.1080/15592294.2016.1273308 28059592 PMC5406215

[149] Pérez-Rodríguez D., López-Fernández H. and Agís-Balboa R.C. (2021) Application of miRNA-seq in neuropsychiatry: a methodological perspective. Comput. Biol. Med. 135, 104603 10.1016/j.compbiomed.2021.104603 34216893

[150] Rotter A., Bayerlein K., Hansbauer M., Weiland J., Sperling W., Kornhuber J. et al. (2013) CB1 and CB2 receptor expression and promoter methylation in patients with cannabis dependence. Eur. Addict. Res. 19, 13–20 10.1159/000338642 22948261

[151] Liu J., Chen J., Ehrlich S., Walton E., White T., Perrone-Bizzozero N. et al. (2014) Methylation patterns in whole blood correlate with symptoms in schizophrenia patients. Schizophr. Bull. 40, 769–776 10.1093/schbul/sbt080 23734059 PMC4059425

[152] DiNieri J.A., Wang X., Szutorisz H., Spano S.M., Kaur J., Casaccia P. et al. (2011) Maternal cannabis use alters ventral striatal dopamine D2 gene regulation in the offspring. Biol. Psychiatry 70, 763–769 10.1016/j.biopsych.2011.06.027 21820648 PMC3186868

[153] Wang X., Dow-Edwards D., Anderson V., Minkoff H. and Hurd Y.L. (2004) In utero marijuana exposure associated with abnormal amygdala dopamine D2 gene expression in the human fetus. Biol. Psychiatry 56, 909–915 10.1016/j.biopsych.2004.10.015 15601599

[154] Ibn Lahmar Andaloussi Z., Taghzouti K. and Abboussi O. (2019) Behavioural and epigenetic effects of paternal exposure to cannabinoids during adolescence on offspring vulnerability to stress. Int. J. Dev. Neurosci. 72, 48–54 10.1016/j.ijdevneu.2018.11.007 30476535

[155] Innocenzi E., De Domenico E., Ciccarone F., Zampieri M., Rossi G., Cicconi R. et al. (2019) Paternal activation of CB2 cannabinoid receptor impairs placental and embryonic growth via an epigenetic mechanism. Sci. Rep. 9, 17034 10.1038/s41598-019-53579-3 31745152 PMC6863860

[156] Sales A.J., Guimarães F.S. and Joca S.R.L. (2020) CBD modulates DNA methylation in the prefrontal cortex and hippocampus of mice exposed to forced swim. Behav. Brain Res. 388, 112627 10.1016/j.bbr.2020.112627 32348868

[157] da Silva V.K., de Freitas B.S., Dornelles V.C., Kist L.W., Bogo M.R., Silva M.C. et al. (2018) Novel insights into mitochondrial molecular targets of iron-induced neurodegeneration: Reversal by cannabidiol. Brain Res. Bull. 139, 1–8 10.1016/j.brainresbull.2018.01.014 29374603

[158] Wanner N.M., Colwell M., Drown C. and Faulk C. (2020) Subacute cannabidiol alters genome-wide DNA methylation in adult mouse hippocampus. Environ. Mol. Mutagen. 61, 890–900 10.1002/em.22396 32579259 PMC7765463

[159] Wu H., Coskun V., Tao J., Xie W., Ge W., Yoshikawa K. et al. (2010) Dnmt3a-dependent nonpromoter DNA methylation facilitates transcription of neurogenic genes. Science 329, 444–448 10.1126/science.1190485 20651149 PMC3539760

[160] Wu Z., Huang K., Yu J., Le T., Namihira M., Liu Y. et al. (2012) Dnmt3a regulates both proliferation and differentiation of mouse neural stem cells. J. Neurosci. Res. 90, 1883–1891 10.1002/jnr.23077 22714992 PMC3418436

[161] García-Gutiérrez M.S., Navarrete F., Gasparyan A., Austrich-Olivares A., Sala F. and Manzanares J. (2020) Cannabidiol: a potential new alternative for the treatment of anxiety, depression, and psychotic disorders. Biomolecules 10, 1–34 10.3390/biom10111575 33228239 PMC7699613

[162] Todd S.M., Zhou C., Clarke D.J., Chohan T.W., Bahceci D. and Arnold J.C. (2017) Interactions between cannabidiol and Δ9-THC following acute and repeated dosing: Rebound hyperactivity, sensorimotor gating and epigenetic and neuroadaptive changes in the mesolimbic pathway. Eur. Neuropsychopharmacol. 27, 132–145 10.1016/j.euroneuro.2016.12.004 28043732

[163] Pastrana-Trejo J.C., Duarte-Aké F., Us-Camas R., De-la-Peña C., Parker L., Pertwee R.G. et al. (2021) Effects on the post-translational modification of H3K4Me3, H3K9ac, H3K9Me2, H3K27Me3, and H3K36Me2 levels in cerebral cortex, hypothalamus and pons of rats after a systemic administration of cannabidiol: a preliminary study. Cent. Nerv. Syst. Agents Med. Chem. 21, 142–147 10.2174/1871524920666200924114524 32972354

[164] Lomazzo E., König F., Abassi L., Jelinek R. and Lutz B. (2017) Chronic stress leads to epigenetic dysregulation in the neuropeptide-Y and cannabinoid CB1 receptor genes in the mouse cingulate cortex. Neuropharmacology 113, 301–313 10.1016/j.neuropharm.2016.10.008 27737789

[165] Wang S.E., Ko S.Y., Kim Y.-S., Jo S., Lee S.H., Jung S.J. et al. (2018) Capsaicin upregulates HDAC2 via TRPV1 and impairs neuronal maturation in mice. Exp. Mol. Med. 50, e455 10.1038/emm.2017.289 29520110 PMC5898893

[166] Wang S.E., Ko S.Y., Jo S., Choi M., Lee S.H., Jo H.-R. et al. (2017) TRPV1 Regulates Stress Responses through HDAC2. Cell Rep. 19, 401–412 10.1016/j.celrep.2017.03.050 28402861

[167] Buran İ., Etem E.Ö., Tektemur A. and Elyas H. (2017) Treatment with TREK1 and TRPC3/6 ion channel inhibitors upregulates microRNA expression in a mouse model of chronic mild stress. Neurosci. Lett. 656, 51–57 10.1016/j.neulet.2017.07.017 28716528

[168] Lee P.H., Perlis R.H., Jung J.Y., Byrne E.M., Rueckert E., Siburian R. et al. (2012) Multi-locus genome-wide association analysis supports the role of glutamatergic synaptic transmission in the etiology of major depressive disorder. Transl. Psychiatry 2, e184 10.1038/tp.2012.95 23149448 PMC3565768

[169] Numakawa T., Nakajima S., Adachi N., Richards M. and Kunugi H. (2013) Neurotrophin Bdnf and novel molecular targets in depression pathogenesis. J. Neurol. Transl. Neurosci. 1, 1–8

[170] Dwivedi Y., Rizavi H.S., Roberts R.C., Conley R.C., Tamminga C.A. and Pandey G.N. (2001) Reduced activation and expression of ERK1/2 MAP kinase in the post-mortem brain of depressed suicide subjects. J. Neurochem. 77, 916–928 10.1046/j.1471-4159.2001.00300.x 11331420

[171] Launay J., Mouillet-Richard S., Baudry A., Pietri M. and Kellermann O. (2011) Raphe-mediated signals control the hippocampal response to SRI antidepressants via miR-16. Transl. Psychiatry 1, e56 10.1038/tp.2011.54 22833211 PMC3309472

[172] Bai M., Zhu X., Zhang Y., Zhang S., Zhang L., Xue L. et al. (2012) Abnormal hippocampal BDNF and miR-16 expression is associated with depression-like behaviors induced by stress during early life. PLoS ONE 7, e46921 10.1371/journal.pone.0046921 23056528 PMC3466179

[173] Tochigi M., Iwamoto K., Bundo M., Sasaki T., Kato N. and Kato T. (2008) Gene expression profiling of major depression and suicide in the prefrontal cortex of postmortem brains. Neurosci. Res. 60, 184–191 10.1016/j.neures.2007.10.010 18068248

[174] Shi Y., Yuan Y., Xu Z., Pu M., Wang C., Zhang Y. et al. (2012) Genetic variation in the calcium/calmodulin-dependent protein kinase (CaMK) pathway is associated with antidepressant response in females. J. Affect. Disord. 136, 558–566 10.1016/j.jad.2011.10.030 22119081

[175] Portugalov A., Zaidan H., Gaisler-Salomon I., Hillard C.J. and Akirav I. (2022) FAAH Inhibition restores early life stress-induced alterations in PFC microRNAs associated with depressive-like behavior in male and female rats. Int. J. Mol. Sci. 23, 1–23 10.3390/ijms232416101 36555739 PMC9782513

[176] Chiarlone A., Börner C., Martín-Gómez L., Jiménez-González A., García-Concejo A., García-Bermejo M.L. et al. (2016) MicroRNA let-7d is a target of cannabinoid CB1 receptor and controls cannabinoid signaling. Neuropharmacology 108, 345–352 10.1016/j.neuropharm.2016.05.007 27179908

[177] Wei Y.B., Liu J.J., Villaescusa J.C., Åberg E., Brené S., Wegener G. et al. (2016) Elevation of Il6 is associated with disturbed let-7 biogenesis in a genetic model of depression. Translational Psychiatry 6, e869 10.1038/tp.2016.136 27529677 PMC5022082

[178] Bahi A. and Dreyer J.-L. (2018) Lentiviral-mediated let-7d microRNA overexpression induced anxiolytic- and anti-depressant-like behaviors and impaired dopamine D3 receptor expression. Eur. Neuropsychopharmacol. 28, 1394–1404 10.1016/j.euroneuro.2018.09.004 30244920

[179] Zhao C., Sun G., Ye P., Li S. and Shi Y. (2013) MicroRNA let-7d regulates the TLX/microRNA-9 cascade to control neural cell fate and neurogenesis. Sci. Rep. 3, 1329 10.1038/srep01329 23435502 PMC3580325

[180] Chandrasekar V. and Dreyer J.-L. (2009) microRNAs miR-124, let-7d and miR-181a regulate Cocaine-induced Plasticity. Mol. Cell. Neurosci. 42, 350–362 10.1016/j.mcn.2009.08.009 19703567

[181] Ferber S.G., Roth T.L. and Weller A. (2020) Epigenetic fragility of the endocannabinoid system under stress: risk for mood disorders and pharmacogenomic implications. Epigenomics 12, 657–660 10.2217/epi-2020-0037 32396405

[182] Salamat J.M., Abbott K.L., Flannery P.C., Ledbetter E.L. and Pondugula S.R. (2022) Interplay between the cannabinoid system and microRNAs in cancer. ACS Omega 7, 9995–10000 10.1021/acsomega.2c00635 35382335 PMC8973111

[183] Meccariello R., Santoro A., Angelo S., Morrone R., Fasano S., Viggiano A. et al. (2020) The epigenetics of the endocannabinoid system. Int. J. Mol. Sci. 21, 10.3390/ijms21031113 32046164 PMC7037698

[184] Gomes T.M., Dias da Silva D., Carmo H., Carvalho F. and Silva J.P. (2020) Epigenetics and the endocannabinoid system signaling: An intricate interplay modulating neurodevelopment. Pharmacol. Res. 162, 105237 10.1016/j.phrs.2020.105237 33053442

[185] Tao R., Li C., Jaffe A.E., Shin J.H., Deep-Soboslay A., Yamin Re et al. (2020) Cannabinoid receptor CNR1 expression and DNA methylation in human prefrontal cortex, hippocampus and caudate in brain development and schizophrenia. Transl. Psychiatry 10, 158 10.1038/s41398-020-0832-8 32433545 PMC7237456

[186] Wang D., Wang H., Ning W., Backlund M.G., Dey S.K. and DuBois R.N. (2008) Loss of cannabinoid receptor 1 accelerates intestinal tumor growth. Cancer Res. 68, 6468–6476 10.1158/0008-5472.CAN-08-0896 18676872 PMC2561258

[187] Xia D., Wang D., Kim S.-H., Katoh H. and DuBois R.N. (2012) Prostaglandin E2 promotes intestinal tumor growth via DNA methylation. Nat. Med. 18, 224–226 10.1038/nm.2608 22270723 PMC3274627

[188] Hong S., Zheng G. and Wiley J.W. (2015) Epigenetic regulation of genes that modulate chronic stress-induced visceral pain in the peripheral nervous system. Gastroenterology 148, 148.e7–157.e7 10.1053/j.gastro.2014.09.032 25263804 PMC4274248

[189] D'Addario C., Micale V., Di Bartolomeo M., Stark T., Pucci M., Sulcova A. et al. (2017) A preliminary study of endocannabinoid system regulation in psychosis: Distinct alterations of CNR1 promoter DNA methylation in patients with schizophrenia. Schizophr. Res. 188, 132–140 10.1016/j.schres.2017.01.022 28108228

[190] Pucci M., Micioni Di Bonaventura M.V., Vezzoli V., Zaplatic E., Massimini M., Mai S. et al. (2019) Preclinical and clinical evidence for a distinct regulation of Mu opioid and Type 1 cannabinoid receptor genes expression in obesity. Front. Genet. 10, 523 10.3389/fgene.2019.00523 31258545 PMC6588048

[191] Mancino S., Burokas A., Gutiérrez-Cuesta J., Gutiérrez-Martos M., Martín-García E., Pucci M. et al. (2015) Epigenetic and proteomic expression changes promoted by eating addictive-like behavior. Neuropsychopharmacology 40, 2788–2800 10.1038/npp.2015.129 25944409 PMC4864655

[192] Di Francesco A., Falconi A., Di Germanio C., Micioni Di Bonaventura M.V., Costa A., Caramuta S. et al. (2015) Extravirgin olive oil up-regulates CB1 tumor suppressor gene in human colon cancer cells and in rat colon via epigenetic mechanisms. J. Nutr. Biochem. 26, 250–258 10.1016/j.jnutbio.2014.10.013 25533906

[193] D'Addario C., Zaplatic E., Giunti E., Pucci M., Micioni Di Bonaventura M.V., Scherma M. et al. (2020) Epigenetic regulation of the cannabinoid receptor CB1 in an activity-based rat model of anorexia nervosa. Int. J. Eat. Disord. 53, 432–446 10.1002/eat.23271 32275093

[194] Börner C., Martella E., Höllt V. and Kraus J. (2012) Regulation of opioid and cannabinoid receptor genes in human neuroblastoma and T cells by the epigenetic modifiers trichostatin A and 5-Aza-2′-deoxycytidine. NeuroImmunoModulation 19, 180–186 10.1159/000331474 22262103

[195] Chen J., Hutchison K.E., Bryan A.D., Filbey F.M., Calhoun V.D., Claus E.D. et al. (2018) Opposite Epigenetic associations with alcohol use and exercise intervention. Front. Psychiatry 9, 594 10.3389/fpsyt.2018.00594 30498460 PMC6249510

[196] D'Addario C., Di Francesco A., Arosio B., Gussago C., Dell'Osso B., Bari M. et al. (2012) Epigenetic regulation of fatty acid amide hydrolase in Alzheimer disease. PLoS ONE 7, e39186 10.1371/journal.pone.0039186 22720070 PMC3373611

[197] Franklin T.B., Russig H., Weiss I.C., Gräff J., Linder N., Michalon A. et al. (2010) Epigenetic transmission of the impact of early stress across generations. Biol. Psychiatry 68, 408–415 10.1016/j.biopsych.2010.05.036 20673872

[198] Uddin M., Aiello A.E., Wildman D.E., Koenen K.C., Pawelec G., de los Santos R. et al. (2010) Epigenetic and immune function profiles associated with posttraumatic stress disorder. Proc. Natl. Acad. Sci. 107, 9470–9475 10.1073/pnas.0910794107 20439746 PMC2889041

[199] Hill M.N., Campolongo P., Yehuda R. and Patel S. (2018) Integrating endocannabinoid signaling and cannabinoids into the biology and treatment of posttraumatic stress disorder. Neuropsychopharmacology 43, 80–102 10.1038/npp.2017.162 28745306 PMC5719095

[200] Subbanna S., Nagre N.N., Umapathy N.S., Pace B.S. and Basavarajappa B.S. (2015) Ethanol exposure induces neonatal neurodegeneration by enhancing CB1R exon1 histone H4K8 acetylation and up-regulating CB1R function causing neurobehavioral abnormalities in adult mice. Int. J. Neuropsychopharmacolog. 18, pyu028 10.1093/ijnp/pyu028 PMC4376538 25609594

[201] Subbanna S., Shivakumar M., Psychoyos D., Xie S. and Basavarajappa B.S. (2013) Anandamide-CB1 receptor signaling contributes to postnatal ethanol-induced neonatal neurodegeneration, adult synaptic, and memory deficits. J. Neurosci.: Off. J. Soc. Neurosci. 33, 6350–6366 10.1523/JNEUROSCI.3786-12.2013 23575834 PMC3742029

[202] Luo Y., Zhang J., Chen L., Chen S.-R., Chen H., Zhang G. et al. (2020) Histone methyltransferase G9a diminishes expression of cannabinoid CB1 receptors in primary sensory neurons in neuropathic pain. J. Biol. Chem. 295, 3553–3562 10.1074/jbc.RA119.011053 32024693 PMC7076223

[203] Pucci M., Micioni Di Bonaventura M.V., Zaplatic E., Bellia F., Maccarrone M., Cifani C. et al. (2019) Transcriptional regulation of the endocannabinoid system in a rat model of binge-eating behavior reveals a selective modulation of the hypothalamic fatty acid amide hydrolase gene. Int. J. Eat. Disord. 52, 51–60 10.1002/eat.22989 30578649

[204] Tung C.W., Ho C., Hsu Y.C., Huang S.C., Shih Y.H. and Lin C.L. (2019) MicroRNA-29a attenuates diabetic glomerular injury through modulating cannabinoid receptor 1 signaling. Molecules 24, 10.3390/molecules24020264 30642005 PMC6359641

[205] Sredni S.T., Huang C.C., Suzuki M., Pundy T., Chou P. and Tomita T. (2016) Spontaneous involution of pediatric low-grade gliomas: high expression of cannabinoid receptor 1 (CNR1) at the time of diagnosis may indicate involvement of the endocannabinoid system. Childs Nervous System 32, 2061–2067 10.1007/s00381-016-3243-7 27613640

[206] Li L., Xu Y., Zhao M. and Gao Z. (2020) Neuro-protective roles of long non-coding RNA MALAT1 in Alzheimer's disease with the involvement of the microRNA-30b/CNR1 network and the following PI3K/AKT activation. Exp. Mol. Pathol. 117, 104545 10.1016/j.yexmp.2020.104545 32976819

[207] Gou X., Wu J., Huang M., Weng Y., Yang T., Chen T. et al. (2020) microRNA-128 mediates CB1 expression and regulates NF-KB/p-JNK axis to influence the occurrence of diabetic bladder disease. J. Transl. Med. 18, 284 10.1186/s12967-020-02406-9 32678046 PMC7367232

[208] Zhang A., Bai Z., Yi W., Hu Z. and Hao J. (2021) Overexpression of miR-338-5p in exosomes derived from mesenchymal stromal cells provides neuroprotective effects by the Cnr1/Rap1/Akt pathway after spinal cord injury in rats. Neurosci. Lett. 761, 136124 10.1016/j.neulet.2021.136124 34302891

[209] Möhnle P., Schütz S.V., Schmidt M., Hinske C., Hübner M., Heyn J. et al. (2014) MicroRNA-665 is involved in the regulation of the expression of the cardioprotective cannabinoid receptor CB2 in patients with severe heart failure. Biochem. Biophys. Res. Commun. 451, 516–521 10.1016/j.bbrc.2014.08.008 25111814

[210] Xu A., Yang Y., Shao Y., Wu M. and Sun Y. (2019) Inhibiting effect of microRNA-187-3p on osteogenic differentiation of osteoblast precursor cells by suppressing cannabinoid receptor type 2. Differentiation 109, 9–15 10.1016/j.diff.2019.07.002 31352121

[211] Most D., Salem N.A., Tiwari G.R., Blednov Y.A., Mayfield R.D. and Harris R.A. (2019) Silencing synaptic MicroRNA-411 reduces voluntary alcohol consumption in mice. Addict. Biol. 24, 604–616 10.1111/adb.12625 29665166 PMC6192878

[212] Huang C., Wang Y., Wu Z., Xu J., Zhou L., Wang D. et al. (2021) miR-98-5p plays a critical role in depression and antidepressant effect of ketamine. Transl. Psychiatry 11, 454 10.1038/s41398-021-01588-0 34480014 PMC8417029

[213] Uchida S., Hara K., Kobayashi A., Funato H., Hobara T., Otsuki K. et al. (2010) Early life stress enhances behavioral vulnerability to stress through the activation of REST4-mediated gene transcription in the medial prefrontal cortex of rodents. J. Neurosci. 30, 15007–15018 10.1523/JNEUROSCI.1436-10.2010 21068306 PMC6633839

[214] Maffioletti E., Bocchio-Chiavetto L., Perusi G., Carvalho Silva R., Sacco C., Bazzanella R. et al. (2021) Inflammation-related microRNAs are involved in stressful life events exposure and in trauma-focused psychotherapy in treatment-resistant depressed patients. Eur. J. Psychotraumatol. 12, 1987655 10.1080/20008198.2021.1987655 35070159 PMC8772504

[215] Honda M., Kuwano Y., Katsuura-Kamano S., Kamezaki Y., Fujita K., Akaike Y. et al. (2013) Chronic academic stress increases a group of microRNAs in peripheral blood. PLoS ONE 8, e75960 10.1371/journal.pone.0075960 24130753 PMC3794012

[216] Sillivan S.E., Jones M.E., Jamieson S., Rumbaugh G. and Miller C.A. (2019) Bioinformatic analysis of long-lasting transcriptional and translational changes in the basolateral amygdala following acute stress. PLoS ONE 14, e0209846 10.1371/journal.pone.0209846 30629705 PMC6328204

[217] Wan Y., Liu Y., Wang X., Wu J., Liu K., Zhou J. et al. (2015) Identification of differential microRNAs in cerebrospinal fluid and serum of patients with major depressive disorder. PLoS ONE 10, e0121975 10.1371/journal.pone.0121975 25763923 PMC4357380

[218] Wan Y.-Q., Feng J.-G., Li M., Wang M.-Z., Liu L., Liu X. et al. (2018) Prefrontal cortex miR-29b-3p plays a key role in the antidepressant-like effect of ketamine in rats. Exp. Mol. Med. 50, 1–14 10.1038/s12276-018-0164-4 PMC6204429 30369596

[219] Rinaldi A., Vincenti S., De Vito F., Bozzoni I., Oliverio A., Presutti C. et al. (2010) Stress induces region specific alterations in microRNAs expression in mice. Behav. Brain Res. 208, 265–269 10.1016/j.bbr.2009.11.012 19913057

[220] Kye M.J., Neveu P., Lee Y.S., Zhou M., Steen J.A., Sahin M. et al. (2011) NMDA mediated contextual conditioning changes miRNA expression. PLoS ONE 6, e24682 10.1371/journal.pone.0024682 21931811 PMC3171446

[221] Gorinski N., Bijata M., Prasad S., Wirth A., Abdel Galil D., Zeug A. et al. (2019) Attenuated palmitoylation of serotonin receptor 5-HT1A affects receptor function and contributes to depression-like behaviors. Nat. Commun. 10, 3924 10.1038/s41467-019-11876-5 31477731 PMC6718429

[222] Pearson-Leary J., Eacret D., Chen R., Takano H., Nicholas B. and Bhatnagar S. (2017) Inflammation and vascular remodeling in the ventral hippocampus contributes to vulnerability to stress. Transl. Psychiatry 7, e1160 10.1038/tp.2017.122 28654094 PMC5537643

[223] Shen M., Song Z. and Wang J.H. (2019) microRNA and mRNA profiles in the amygdala are associated with stress-induced depression and resilience in juvenile mice. Psychopharmacology (Berl.) 236, 2119–2142 10.1007/s00213-019-05209-z 30900007

[224] Martin C.G., Kim H., Yun S., Livingston W., Fetta J., Mysliwiec V. et al. (2017) Circulating miRNA associated with posttraumatic stress disorder in a cohort of military combat veterans. Psychiatry Res. 251, 261–265 10.1016/j.psychres.2017.01.081 28222310 PMC6065100

[225] Roy B., Dunbar M., Agrawal J., Allen L. and Dwivedi Y. (2020) Amygdala-based altered miRNome and epigenetic contribution of miR-128-3p in conferring susceptibility to depression-like behavior via Wnt signaling. Int. J. Neuropsychopharmacol. 23, 165–177 10.1093/ijnp/pyz071 32173733 PMC7171932

[226] Lin Q., Wei W., Coelho C.M., Li X., Baker-Andresen D., Dudley K. et al. (2011) The brain-specific microRNA miR-128b regulates the formation of fear-extinction memory. Nat. Neurosci. 14, 1115–1117 10.1038/nn.2891 21841775

[227] Smalheiser N.R., Lugli G., Rizavi H.S., Torvik V.I., Turecki G. and Dwivedi Y. (2012) MicroRNA expression is down-regulated and reorganized in prefrontal cortex of depressed suicide subjects. PloS ONE 7, e33201 10.1371/journal.pone.0033201 22427989 PMC3302855

[228] Sun X., Song Z., Si Y. and Wang J.-H. (2018) microRNA and mRNA profiles in ventral tegmental area relevant to stress-induced depression and resilience. Prog. Neuropsychopharmacol. Biol. Psychiatry 86, 150–165 10.1016/j.pnpbp.2018.05.023 29864451

[229] Yang J., Sun J., Lu Y., An T., Lu W. and Wang J.H. (2020) Revision to psychopharmacology mRNA and microRNA profiles are associated with stress susceptibility and resilience induced by psychological stress in the prefrontal cortex. Psychopharmacology (Berl.) 237, 3067–3093 10.1007/s00213-020-05593-x 32591938

[230] Homorogan C., Enatescu V.R., Nitusca D., Marcu A., Seclaman E. and Marian C. (2021) Distribution of microRNAs associated with major depressive disorder among blood compartments. J. Int. Med. Res. 49, 3000605211006633 10.1177/03000605211006633 33827323 PMC8040584

[231] Teppen T.L., Krishnan H.R., Zhang H., Sakharkar A.J. and Pandey S.C. (2016) The potential role of amygdaloid microRNA-494 in alcohol-induced anxiolysis. Biol. Psychiatry 80, 711–719 10.1016/j.biopsych.2015.10.028 26786313 PMC4882267

[232] Belzeaux R., Bergon A., Jeanjean V., Loriod B., Formisano-Tréziny C., Verrier L. et al. (2012) Responder and nonresponder patients exhibit different peripheral transcriptional signatures during major depressive episode. Transl. Psychiatry 2, e185 10.1038/tp.2012.112 23149449 PMC3565773

[233] Balakathiresan N.S., Chandran R., Bhomia M., Jia M., Li H. and Maheshwari R.K. (2014) Serum and amygdala microRNA signatures of posttraumatic stress: fear correlation and biomarker potential. J. Psychiatr. Res. 57, 65–73 10.1016/j.jpsychires.2014.05.020 24998397

[234] Solich J., Kolasa M., Faron-Górecka A., Hajto J., Piechota M. and Dziedzicka-Wasylewska M. (2021) MicroRNA Let-7e in the mouse prefrontal cortex differentiates restraint-stress-resilient genotypes from susceptible genotype. Int. J. Mol. Sci. 22, 1–16 10.3390/ijms22179439 PMC8430919 34502349

[235] Maffioletti E., Cattaneo A., Rosso G., Maina G., Maj C., Gennarelli M. et al. (2016) Peripheral whole blood microRNA alterations in major depression and bipolar disorder. J. Affect. Disord. 200, 250–258 10.1016/j.jad.2016.04.021 27152760

[236] Maurel O.M., Torrisi S.A., Barbagallo C., Purrello M., Salomone S., Drago F. et al. (2021) Dysregulation of miR-15a-5p, miR-497a-5p and miR-511-5p is associated with modulation of BDNF and FKBP5 in brain areas of PTSD-related susceptible and resilient mice. Int. J. Mol. Sci. 22, 10.3390/ijms22105157 PMC8153003 34068160

[237] Zhao C., Zhou B., Cao J., Zhang Y., Li W., Wang M. et al. (2020) miR-187-3p participates in contextual fear memory formation through modulating SATB2 expression in the hippocampus. Neuroreport 31, 909–917 10.1097/WNR.0000000000001484 32568775

[238] Su J., Li P., Zhuang Q., Chen X., Zhang X., Li X. et al. (2021) Identification of the similarities and differences of molecular networks associated with fear memory formation, extinction, and updating in the amygdala. Front. Mol. Neurosci. 14, 778170 10.3389/fnmol.2021.778170 34924954 PMC8675638

[239] McKibben L.A. and Dwivedi Y. (2021) Early life and adult stress promote sex dependent changes in hypothalamic miRNAs and environmental enrichment prevents stress-induced miRNA and gene expression changes in rats. BMC Genomics 22, 701 10.1186/s12864-021-08003-4 34583641 PMC8480023

[240] McKibben L.A. and Dwivedi Y. (2021) Early-life stress induces genome-wide sex-dependent miRNA expression and correlation across limbic brain areas in rats. Epigenomics 13, 1031–1056 10.2217/epi-2021-0037 34008410 PMC8244583

[241] Patrício P., Mateus-Pinheiro A., Irmler M., Alves N.D., Machado-Santos A.R., Morais M. et al. (2015) Differential and converging molecular mechanisms of antidepressants' action in the hippocampal dentate gyrus. Neuropsychopharmacology 40, 338–349 10.1038/npp.2014.176 25035085 PMC4443946

[242] Gupta S., Kim S.Y., Artis S., Molfese D.L., Schumacher A., Sweatt J.D. et al. (2010) Histone methylation regulates memory formation. J. Neurosci.:Off. J. Soc. Neurosci. 30, 3589–3599 10.1523/JNEUROSCI.3732-09.2010 20219993 PMC2859898

[243] Peixoto L. and Abel T. (2013) The role of histone acetylation in memory formation and cognitive impairments. Neuropsychopharmacology 38, 62–76 10.1038/npp.2012.86 22669172 PMC3521994

[244] Ookubo M., Kanai H., Aoki H. and Yamada N. (2013) Antidepressants and mood stabilizers effects on histone deacetylase expression in C57BL/6 mice: Brain region specific changes. J. Psychiatr. Res. 47, 1204–1214 10.1016/j.jpsychires.2013.05.028 23777937

[245] Manzanares J., Cabañero D., Puente N., García-Gutiérrez M.S., Grandes P. and Maldonado R. (2018) Role of the endocannabinoid system in drug addiction. Biochem. Pharmacol. 157, 108–121 10.1016/j.bcp.2018.09.013 30217570

[246] Wolfe S.A., Vozella V. and Roberto M. (2022) The synaptic interactions of alcohol and the endogenous cannabinoid system. Alcohol Res.: Curr. Rev. 42, 03 10.35946/arcr.v42.1.03 35223337 PMC8843413

[247] Gobira P.H., Joca S.R. and Moreira F.A. (2022) Roles of cannabinoid CB1 and CB2 receptors in the modulation of psychostimulant responses. Acta Neuropsychiatrica 1–11 10.1017/neu.2022.23 35993329

[248] Mantsch J.R., Baker D.A., Funk D., Le A.D. and Shaham Y. (2016) Stress-induced reinstatement of drug seeking: 20 years of progress. Neuropsychopharmacology 41, 335–356 10.1038/npp.2015.142 25976297 PMC4677117

[249] Bardo M.T., Hammerslag L.R. and Malone S.G. (2021) Effect of early life social adversity on drug abuse vulnerability: focus on corticotropin-releasing factor and oxytocin. Neuropharmacology 191, 108567 10.1016/j.neuropharm.2021.108567 33862030 PMC8217369

[250] Tung L.W., Lu G.L., Lee Y.H., Yu L., Lee H.J., Leishman E. et al. (2016) Orexins contribute to restraint stress-induced cocaine relapse by endocannabinoid-mediated disinhibition of dopaminergic neurons. Nat. Commun. 7, 12199 10.1038/ncomms12199 27448020 PMC4961842

[251] McReynolds J.R., Doncheck E.M., Li Y., Vranjkovic O., Graf E.N., Ogasawara D. et al. (2018) Stress promotes drug seeking through glucocorticoid-dependent endocannabinoid mobilization in the prelimbic cortex. Biol. Psychiatry 84, 85–94 10.1016/j.biopsych.2017.09.024 29100630 PMC5889367

[252] Sinha R (2008) Chronic Stress, Drug Use, and Vulnerability to Addiction. Annals of the New York Academy of Sciences 1141, 1 105–130 10.1196/annals.1441.030 18991954 PMC2732004

[253] De Sa Nogueira D., Bourdy R., Alcala-Vida R., Filliol D., Andry V., Goumon Y. et al. (2022) Hippocampal cannabinoid 1 receptors are modulated following cocaine self-administration in male rats. Mol. Neurobiol. 59, 1896–1911 10.1007/s12035-022-02722-9 35032317

[254] Subbanna S., Nagre N.N., Umapathy N.S., Pace B.S. and Basavarajappa B.S. (2014) Ethanol exposure induces neonatal neurodegeneration by enhancing CB1R Exon1 histone H4K8 acetylation and up-regulating CB1R function causing neurobehavioral abnormalities in adult mice. Int. J. Neuropsychopharmacol. 18, 1–15 10.1093/ijnp/pyu028 25609594 PMC4376538

[255] Nagre N.N., Subbanna S., Shivakumar M., Psychoyos D. and Basavarajappa B.S. (2015) CB1-receptor knockout neonatal mice are protected against ethanol-induced impairments of DNMT1, DNMT3A, and DNA methylation. J. Neurochem. 132, 429–442 10.1111/jnc.13006 25487288 PMC4351764

[256] Shivakumar M., Subbanna S., Joshi V. and Basavarajappa B.S. (2020) Postnatal ethanol exposure activates HDAC-mediated histone deacetylation, impairs synaptic plasticity gene expression and behavior in mice. Int. J. Neuropsychopharmacol. 23, 324–338 10.1093/ijnp/pyaa017 32170298 PMC7251635

[257] Subbanna S., Nagre N.N., Shivakumar M., Joshi V., Psychoyos D., Kutlar A. et al. (2018) CB1R-mediated activation of caspase-3 causes epigenetic and neurobehavioral abnormalities in postnatal ethanol-exposed mice. Front Mol. Neurosci. 11, 45 10.3389/fnmol.2018.00045 29515368 PMC5826222

[258] Stringer R.L., Laufer B.I., Kleiber M.L. and Singh S.M. (2013) Reduced expression of brain cannabinoid receptor 1 (Cnr1) is coupled with an increased complementary micro-RNA (miR-26b) in a mouse model of fetal alcohol spectrum disorders. Clin. Epigenetics 5, 14 10.1186/1868-7083-5-14 23915435 PMC3751098

[259] Szutorisz H. and Hurd Y.L. (2018) High times for cannabis: Epigenetic imprint and its legacy on brain and behavior. Neurosci. Biobehav. Rev. 85, 93–101 10.1016/j.neubiorev.2017.05.011 28506926 PMC5682234

[260] Tomasiewicz H.C., Jacobs M.M., Wilkinson M.B., Wilson S.P., Nestler E.J. and Hurd Y.L. (2012) Proenkephalin mediates the enduring effects of adolescent cannabis exposure associated with adult opiate vulnerability. Biol. Psychiatry 72, 803–810 10.1016/j.biopsych.2012.04.026 22683090 PMC3440551

[261] Prini P., Penna F., Sciuccati E., Alberio T. and Rubino T. (2017) Chronic Δ8-THC exposure differently affects histone modifications in the adolescent and adult rat brain. Int. J. Mol. Sci. 18, 10.3390/ijms18102094 28976920 PMC5666776

[262] Prini P., Rusconi F., Zamberletti E., Gabaglio M., Penna F., Fasano M. et al. (2018) Adolescent THC exposure in female rats leads to cognitive deficits through a mechanism involving chromatin modifications in the prefrontal cortex. J. Psychiatry Neurosci.:JPN 43, 87–101 10.1503/jpn.170082 29481316 PMC5837889

[263] Watson C.T., Szutorisz H., Garg P., Martin Q., Landry J.A., Sharp A.J. et al. (2015) Genome-wide DNA methylation profiling reveals epigenetic changes in the rat nucleus accumbens associated with cross-generational effects of adolescent THC exposure. Neuropsychopharmacology 40, 2993–3005 10.1038/npp.2015.155 26044905 PMC4864634

[264] Levin E.D., Hawkey A.B., Hall B.J., Cauley M., Slade S., Yazdani E. et al. (2019) Paternal THC exposure in rats causes long-lasting neurobehavioral effects in the offspring. Neurotoxicol. Teratol. 74, 106806 10.1016/j.ntt.2019.04.003 31028824

[265] Murphy S.K., Itchon-Ramos N., Visco Z., Huang Z., Grenier C., Schrott R. et al. (2018) Cannabinoid exposure and altered DNA methylation in rat and human sperm. Epigenetics 13, 1208–1221 10.1080/15592294.2018.1554521 30521419 PMC6986792

[266] Schrott R., Rajavel M., Acharya K., Huang Z., Acharya C., Hawkey A. et al. (2020) Sperm DNA methylation altered by THC and nicotine: Vulnerability of neurodevelopmental genes with bivalent chromatin. Sci. Rep. 10, 16022 10.1038/s41598-020-72783-0 32994467 PMC7525661

[267] Schrott R., Greeson K.W., King D., Symosko Crow K.M., Easley C.At. and Murphy S.K. (2022) Cannabis alters DNA methylation at maternally imprinted and autism candidate genes in spermatogenic cells. Systems Biol. Reproduct. Med. 68, 357–369 10.1080/19396368.2022.2073292 PMC10032331 35687495

[268] D'Addario C., Di Francesco A., Pucci M., Finazzi Agrò A. and Maccarrone M. (2013) Epigenetic mechanisms and endocannabinoid signalling. FEBS J. 280, 1905–1917 10.1111/febs.12125 23305292

[269] Rusconi F., Rubino T. and Battaglioli E. (2020) Endocannabinoid-epigenetic cross-talk: a bridge toward stress coping. Int. J. Mol. Sci. 21, 1–13 10.3390/ijms21176252 32872402 PMC7504015

